# Metabolic Profiling of Adiponectin Levels in Adults: Mendelian Randomization Analysis

**DOI:** 10.1161/CIRCGENETICS.117.001837

**Published:** 2017-12-13

**Authors:** Maria Carolina Borges, Aluísio J.D. Barros, Diana L. Santos Ferreira, Juan Pablo Casas, Bernardo Lessa Horta, Mika Kivimaki, Meena Kumari, Usha Menon, Tom R. Gaunt, Yoav Ben-Shlomo, Deise F. Freitas, Isabel O. Oliveira, Aleksandra Gentry-Maharaj, Evangelia Fourkala, Debbie A. Lawlor, Aroon D. Hingorani

**Affiliations:** From the Post-Graduate Program in Epidemiology, Federal University of Pelotas, Brazil (M.C.B., A.J.D.B., B.L.H., D.F.F., I.O.O.); MRC Integrative Epidemiology Unit (M.C.B., D.L.S.F., T.R.G., D.A.L.) and Population Health Sciences, Bristol Medical School (M.C.B., D.L.S.F., T.R.G., Y.B.-S., D.A.L.), University of Bristol, United Kingdom; Farr Institute of Health Informatics (J.P.C., A.D.H.), Department of Epidemiology and Public Health (M. Kivimaki, M. Kumari), Department of Women’s Cancer, Institute for Women’s Health, Faculty of Population Health Sciences (U.M., A.G.-M., E.F.), and Institute of Cardiovascular Science (A.D.H.), University College London, United Kingdom; Institute for Social and Economic Research, University of Essex, United Kingdom (M. Kumari); and Department of Physiology and Pharmacology, Institute of Biology, Federal University of Pelotas, Brazil (I.O.O.).

**Keywords:** adiponectin, cardiovascular disease, insulin, Mendelian Randomization Analysis, metabolism, metabolomics

## Abstract

**Background—:**

Adiponectin, a circulating adipocyte-derived protein, has insulin-sensitizing, anti-inflammatory, antiatherogenic, and cardiomyocyte-protective properties in animal models. However, the systemic effects of adiponectin in humans are unknown. Our aims were to define the metabolic profile associated with higher blood adiponectin concentration and investigate whether variation in adiponectin concentration affects the systemic metabolic profile.

**Methods and Results—:**

We applied multivariable regression in ≤5909 adults and Mendelian randomization (using *cis*-acting genetic variants in the vicinity of the adiponectin gene as instrumental variables) for analyzing the causal effect of adiponectin in the metabolic profile of ≤37 545 adults. Participants were largely European from 6 longitudinal studies and 1 genome-wide association consortium. In the multivariable regression analyses, higher circulating adiponectin was associated with higher high-density lipoprotein lipids and lower very-low-density lipoprotein lipids, glucose levels, branched-chain amino acids, and inflammatory markers. However, these findings were not supported by Mendelian randomization analyses for most metabolites. Findings were consistent between sexes and after excluding high-risk groups (defined by age and occurrence of previous cardiovascular event) and 1 study with admixed population.

**Conclusions—:**

Our findings indicate that blood adiponectin concentration is more likely to be an epiphenomenon in the context of metabolic disease than a key determinant.

## Abstract 2

Supplemental Digital Content is available in the text.

The recognition that adipose tissue is an endocrine organ raised new prospects for discovering adipose-derived products that could be valuable drug targets for the treatment and prevention of cardiometabolic diseases. In this context, adiponectin, a 30 kDa protein largely produced by mature adipocytes, has been attracting widespread attention because of insulin-sensitizing, anti-inflammatory, antiatherogenic, and cardiomyocyte-protective properties demonstrated in animal models.^1^

**See Clinical Perspective**

However, human studies have yielded a far more complicated picture. Unlike most other adipokines, circulating adiponectin concentration is higher with lower adiposity.^2^ In prospective observational studies in humans using multivariable regression, higher circulating adiponectin is associated with lower risk of type 2 diabetes mellitus,^3^ hepatic dysfunction,^4^ and metabolic syndrome^5^ but higher mortality in patients with kidney disease, heart failure, previous cardiovascular disease, or general elderly cohorts^6–9^; this different direction of effect between risk of incident disease and mortality among high-risk groups has been called the adiponectin paradox.^10^

Given the complex metabolic derangements that might participate in and compensatory changes that might occur in response to human diseases, the association between adiponectin concentration and cardiometabolic biomarkers and disease end points might be explained by reverse causality (where disease status could alter adiponectin concentration) or residual confounding (where adiponectin could be a marker of another causal factor, such as adiposity or insulin resistance).^11^ Classical multivariable regression studies cannot distinguish causal from noncausal associations, and randomized controlled trials specifically targeting adiponectin are not possible in the absence of a specific therapeutic targeting adiponectin concentration or function.

Mendelian randomization uses genetic variants (mostly single-nucleotide polymorphisms [SNPs]) that are robustly related to the risk factor of interest as tools to assess its role in causing disease.^12^ The random allocation of parental alleles at meiosis should theoretically reduce confounding in genetic association studies, and this has been shown to be the case^13^; the unidirectional flow of biological information from genetic variant to phenotypes avoids reverse causality. Mendelian randomization has been used in clinical research to investigate potential etiologic mechanisms, such as the causal effects of low-density lipoprotein cholesterol (LDL-C),^14^ systolic blood pressure,^15^ and CRP (C-reactive protein)^16^ on coronary heart disease, validate and prioritize novel drug targets, such as IL-6 (interleukin-6) receptor,^17^ and increase understanding of current therapies, for example, statins.^18^

Previous Mendelian randomization studies indicate that circulating adiponectin is a consequence of low insulin sensitivity,^19^ but whether adiponectin concentration is also a cause of insulin sensitivity is uncertain.^19–21^ Using Mendelian randomization in a study of 63 746 coronary heart disease cases and 130 681 controls, we have recently shown that adiponectin may not be causally related to coronary heart disease.^22^ Although multivariable analyses show that higher adiponectin concentration is associated with lower glycated hemoglobin, insulin, triglycerides (TG), and higher high-density lipoprotein cholesterol (HDL-C), using Mendelian randomization, we found little evidence that these were causal.^22^ Whether adiponectin is associated with systemic metabolic profile, and, if it is, what aspects of these associations are causal is unknown. A broader interrogation of the metabolic effects of adiponectin through high-throughput profiling of metabolic status could provide valuable insights into whether adiponectin is a noncausal biomarker or causally important in the pathophysiology of some human diseases.^23^

We combined genotype, adiponectin, and metabolomics profile data from 6 longitudinal studies and 1 genome-wide association consortium with the aim of (1) defining the metabolic signature of blood adiponectin concentration and (2) investigating whether variation in adiponectin concentration is causally related to the systemic metabolic profile.

## Methods

### Study Populations

The metabolic profile associated with blood adiponectin concentration was examined from 7 data sources: PEL82 (the 1982 Pelotas Birth Cohort), including adults aged 30 years old born in the city of Pelotas, Brazil, in 1982^24,25^; BWHHS (the British Women’s Heart and Health Study), including UK women aged 60 to 79 years old at recruitment in 2000^26^; WHII study (the Whitehall II), including UK government workers aged 45 to 69 years at phase 5 clinical assessment in 1997 to 1999^27^; the CaPS (Caerphilly Prospective Study), including men aged 52 to 72 years at phase III in 1989 to 1993^28^; a case–control study nested in UKCTOCS (the United Kingdom Collaborative Trial of Ovarian Cancer Screening), including UK postmenopausal women aged 50 to 74 years at recruitment in 2001 to 2005^29^; the ALSPAC-M (Cohort of Mothers From the Avon Longitudinal Study of Children and Parents), including UK women aged 34 to 63 years old at clinical assessment in 2009 to 2011^30^; and a metabolomics genome-wide association consortium (hereafter referred to as Metabolomics consortium), including European adults with mean age of 45 years old from 14 cohorts.^31^ Individual-level data were available to investigators from PEL82, BWHHS, WHII, CaPS, UKCTOCS, and ALSPAC-M. Individual-level study data cannot be made available to other researchers for purposes of reproducing the results or replicating the procedure. Summary-level data are publicly available from the Metabolomics consortium (URL: http://www.computationalmedicine.fi/data/NMR_GWAS/).

**Figure 1.**
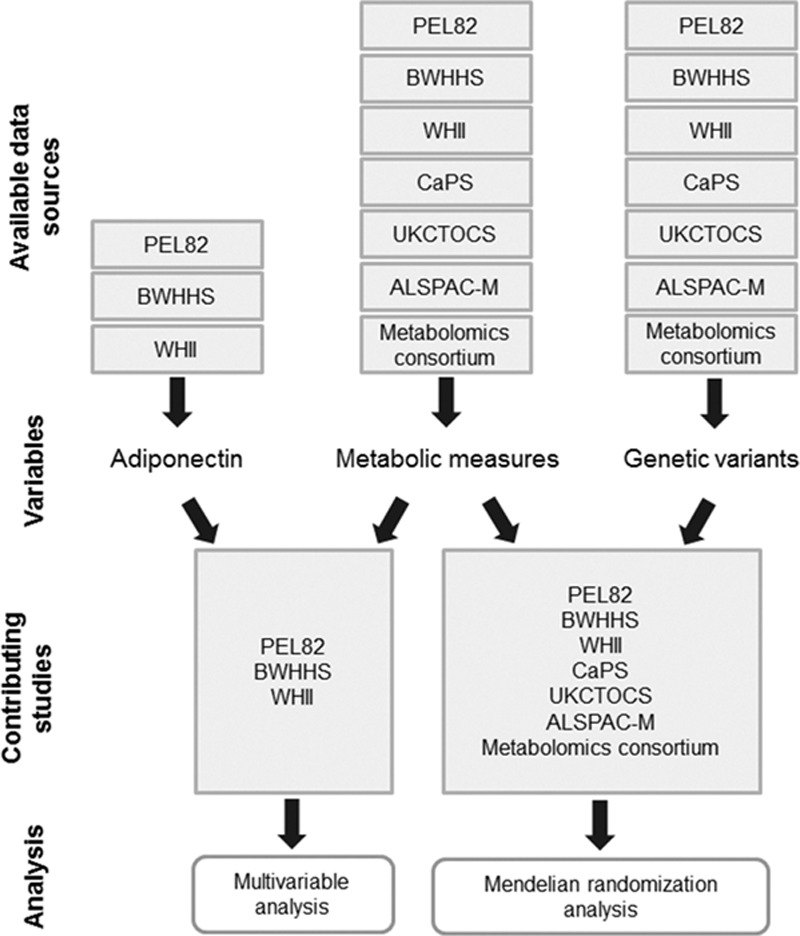
Schematic representation of studies contributing to each analytic approach. From the available data sources, 3 had data on adiponectin and metabolic measures and could contribute to multivariable analysis (PEL82 [1982 Pelotas Birth Cohort], BWHHS [British Women’s Heart and Health Study], and WHII study [Whitehall II]), and all had data on genetic variants and metabolic measures and could contribute to Mendelian randomization analysis (PEL82, BWHHS, WHII, CaPS [the Caerphilly Prospective Study], UKCTOCS [the United Kingdom Collaborative Trial of Ovarian Cancer Screening], ALSPAC-M [Cohort of Mothers From the Avon Longitudinal Study of Children and Parents], and Metabolomics consortium).

All study participants provided written informed consent, and study protocols were approved by the local ethics committees (ethical approval for ALSPAC was also obtained from the ALSPAC Ethics and Law Committee). Studies’ characteristics are summarized in Table 1. We examined (possibly causal) associations of adiponectin with systemic metabolic profiles using 2 approaches—conventional multivariable regression and Mendelian randomization analyses. Studies must have both adiponectin and measures of some of the outcomes (but do not need genetic data) to contribute to multivariable regression analyses and must have relevant genetic variants and outcomes (but do not need adiponectin concentration data) to contribute to Mendelian randomization analyses. Figure 1 shows how the different data sources contributed to the 2 approaches.

**Table 1.**
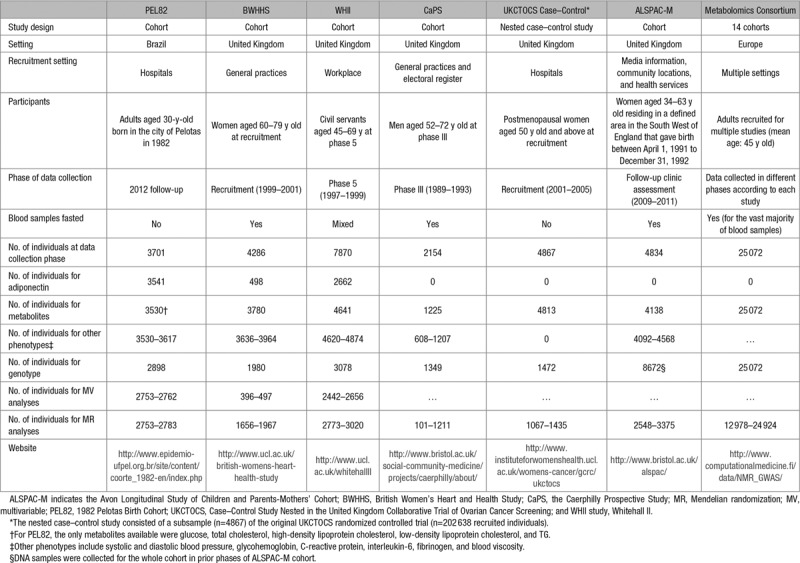
Characteristics of Participating Studies

### Metabolite Quantification

A high-throughput serum nuclear magnetic resonance (NMR) spectroscopy platform was used to quantify ≤150 metabolic measures and 83 derived measures (ratios) in each study. The experimental protocols, including sample preparation and NMR spectroscopy methods, have been described in detail elsewhere^32,33^ and are described briefly in Methods in the Data Supplement. Sixty-six of 150 metabolic measures were selected for this study aimed at broadly representing the systemic metabolite profile, as previously reported by Würtz et al,^34^ including lipoprotein traits (lipid content, particle size, and Apo [apolipoproteins]), fatty acids, amino acids, glycolysis-related metabolites, ketone bodies, fluid balance (albumin and creatinine), and inflammatory markers (glycoprotein acetyls). The remaining 84 metabolic measures from the NMR platform are related to other lipid fractions (esterified and free cholesterol, total cholesterol, TG, and phospholipids) and particle concentration from 14 lipoprotein subclasses. As these 84 metabolic measures are highly correlated with ≥1 of the 66 selected metabolic measures, they were not included in the main analysis (as they would not bring additional information) and were presented in the Data Supplement. Eight additional measures, not obtained from the NMR platform, were included: CRP, IL-6, fibrinogen, blood viscosity, insulin, glycated hemoglobin, and systolic blood pressure and diastolic blood pressure. PEL82 did not have data on metabolic measures from NMR platform and contributed data to analyses of conventional lipid risk factors (total cholesterol, HDL-C, LDL-C, and TG) and some of the additional measures described (CRP, glycated hemoglobin, systolic blood pressure, and diastolic blood pressure). Adiponectin was assayed using an ELISA in PEL82, BWHHS, and WHII. Data on adiponectin level were not available from CaPS, UKCTOCS, ALSPAC-M, and the Metabolomics consortium. Blood samples used for adiponectin, NMR metabolites, and other blood-based outcomes assays were taken after overnight or minimum 6-hour fast in BWHHS, CaPS, and ALSPAC-M and on nonfasting samples in PEL82 and UKCTOCS. In WHII, participants attending the morning clinic were asked to fast overnight and those attending in the afternoon were asked to have a light, fat-free breakfast before 08:00 hours. The vast majority of samples contributing to the Metabolomics consortium were fasting samples.

### Genotyping

BWHHS, CaPS, WHII, and UKCTOCS participants were genotyped using Metabochip, a platform comprising 200 000 SNPs, which cover the loci identified by genome-wide association studies in cardiometabolic diseases and rare variants from the 1000 Genomes Project.^35^ Quality control criteria and imputation using 1000 Genomes European ancestry reference samples have been previously described for studies within UCLEB consortium (University College London, London School of Hygiene & Tropical Medicine, University of Edinburgh and University of Bristol).^36^ In ALSPAC-M, 557 124 SNPs were directly genotyped using Illumina human660W quad. For quality control, SNPs were excluded if missingness >5%, Hardy–Weinberg equilibrium *P* value <1×10^−^^6^, or minor allele frequency <1%, and samples were excluded if missingness >5%, indeterminate X chromosome heterozygosity, extreme autosomal heterozygosity, or showing evidence of population stratification. Imputation was performed using 1000 genomes reference panel (Phase 1, Version 3; phased using ShapeIt v2.r644, haplotype release date December 2013) and Impute V2.2.2. For PEL82, genotyping was performed by using the Illumina HumanOmni2.5-8v1 array (Illumina Inc), and ≈2 500 000 SNPs were genotyped.^37^ For PEL82, quality control criteria have been previously described,^37^ and imputation was performed in 2 steps: first, genotypes were phased using SHAPEIT; then, IMPUTE2 was used for the actual imputation. For autosomal and X chromosome SNPs, 1000 Genomes Phase I integrated haplotypes (December 2013 release) and 1000 Genomes Phase I integrated variant set (March 2012 release), respectively, were used. For PEL82, ancestry-informative principal components were based on 370 539 SNPs shared by samples from the HapMap Project, the Human Genome Diversity Project, and PEL82.^38^ Cohorts contributing to the Metabolomics consortium used different SNP arrays; nongenotyped SNPs were imputed using a 1000 Genomes Project March 2012 version and SNPs with accurate imputation (proper info >0.4) and minor allele count >3 were combined in fixed-effects meta-analysis using double genomic control correction. Further details can be found in the consortium publication.^31^

### Other Covariates

Anthropometric variables (weight and height) were measured in each study using standard procedures, and body mass index was calculated as weight (kg)/height (m)^2^. Demographic and smoking status information was obtained through questionnaires.

### Data Analysis

Before multivariable and genetic analyses, each study adjusted metabolic measures for age, sex, and, if applicable, place of recruitment (BWHHS and UKCTOCS) or principal components of genomic ancestry (PEL82 and some studies contributing to Metabolomics consortium), and the resulting residuals were transformed to normal distribution and standardized using inverse rank-based normal transformation. Pregnant women from PEL82 (n=73) and ALSPAC-M (n=12) were excluded. As the 74 analyzed metabolic measures are highly correlated, we adopted a similar strategy to the Metabolomics consortium^31^ to correct for multiple testing by estimating the number of independent tests as the number of principal components that explained over 95% of variance in metabolic measures using data from the 2 studies (BWHHS and WHII) with the largest available number of metabolites (n=27 principal components in both studies). As a result, for both multivariable and Mendelian randomization analyses, we corrected for multiple testing using the Bonferroni method considering 27 independent tests (*P*=0.05/27≈0.0019). Analyses were conducted in Stata version 12.

#### Multivariable Regression Analysis

The conventional multivariable regression association of adiponectin with individual metabolites was estimated using a 2-stage individual participant meta-analysis. In the first stage, linear regression models were fitted for each study. In the second stage, study-specific estimates were meta-analyzed using DerSimonian and Laird random-effect model.^39^ Heterogeneity across studies was assessed using *I*^2^ (as a measure of the relative size of between-study variation and within-study error).^40^ Three types of subgroup analyses were conducted: sex-stratified analysis, analysis excluding individuals with high risk of cardiometabolic disease (those that had experienced coronary artery disease or stroke or those older than 65 years), and analysis restricted to European studies (excluding PEL82).

#### Genetic Analyses

Four independent SNPs in the vicinity of *ADIPOQ* locus (±50 kb), previously identified to predict adiponectin levels, were selected^22,41^ (details in Methods in the Data Supplement). These SNPs (rs6810075, rs16861209, rs17366568, and rs3774261) are estimated to explain ≈4% of variance in adiponectin concentration (details in Methods in the Data Supplement). Data for the association of each selected SNP with adiponectin concentration in the discovery sample of ADIPOGen, the largest consortium of genome-wide association studies for adiponectin, were downloaded from https://www.mcgill.ca/genepi/adipogen-consortium.

##### Association of Genetic Variants With Classical Confounders

The association between genetic variants and classical confounders (sex, age, ancestry [European versus non-European], current smoking [yes versus no], and body mass index) was examined for each study that provided individual-level data using logistic or linear regression models for binary or continuous variables, respectively.

##### Mendelian Randomization Analysis

To allow all participants with relevant genetic and metabolic measure data to contribute to analyses, even when adiponectin data were not available (as in CaPS, UKCTOCS, ALSPAC-M, and Metabolomics consortium), a 2-sample Mendelian randomization design was used, in which data for the association between genetic variants and adiponectin levels were obtained from an external data source, the ADIPOGen consortium.^42^ The 2-sample Mendelian randomization is a recent extension to the more conventional 1-sample Mendelian randomization and, when samples are independent, has the additional advantage of avoiding bias because of genetic variants correlating with confounders by chance (statistical overfitting).^43^ The 2-sample Mendelian randomization estimates and respective SEs were obtained by combining SNP-specific Wald ratios, as described by Burgess et al^44^ and detailed in Methods in the Data Supplement. Study-specific Mendelian randomization estimates were meta-analyzed using DerSimonian and Laird random-effect model.^39^ Heterogeneity across studies was assessed using *I*^2^.^40^ Subgroup analyses were conducted considering individual-level (sex and risk of cardiometabolic disease) and study-level characteristics (European versus non-European studies). The Metabolomics consortium did not contribute to subgroup analysis of individual-level characteristics as only summary data were available.

#### Comparison Between Multivariable and Mendelian Randomization Analyses

Results from conventional multivariable and Mendelian randomization analyses for each metabolic measure were compared using the Z test (details in the Methods in the Data Supplement) and by estimating the correlation between multivariable and Mendelian randomization estimates across all metabolic measures. Power calculations for multivariable and Mendelian randomization analysis are available in Table I in the Data Supplement.

## Results

The study included a median sample size of 3008 adults in the multivariable analysis (range: 2470–5909) and a median sample size of 29 146 adults in the Mendelian randomization analysis (range: 4647–37 545). Total sample size for each metabolite in multivariable and Mendelian randomization analysis can be found in Table II in the Data Supplement. Characteristics of participants and distribution of metabolites from each contributing study are listed in Table 2 and Table III in the Data Supplement.

**Table 2.**
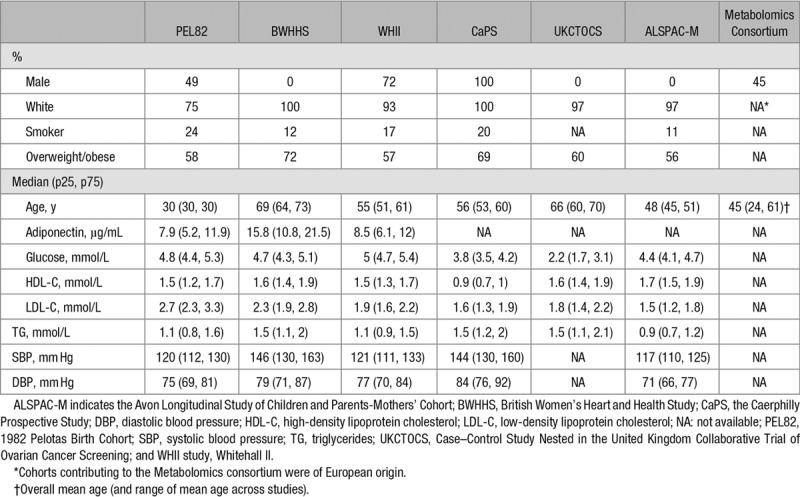
Characteristics of Studies’ Populations

### Adiponectin and the Systemic Metabolic Profile

**Figure 2.**
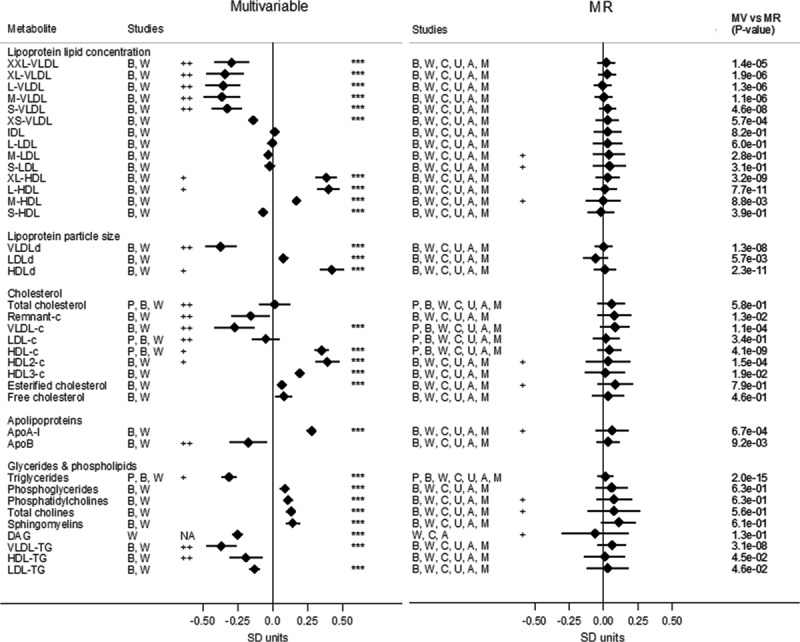
Association of lipoprotein traits with blood adiponectin levels from multivariable and Mendelian randomization (MR) analysis. Values are expressed as units of standardized metabolite concentration (and 95% CI [confidence interval]) per 1 U increment of standardized log adiponectin levels. *P* values for the association between adiponectin and metabolites are indicated by *** if lower than Bonferroni-adjusted threshold (*P* value <0.0019). Heterogeneity was considered substantial if *I*^2^=50% to 75% (+) or high if *I*^2^>75% (++). *P* values for the comparison between multivariable and MR estimates are displayed in the column MR vs MV (*P* value). Metabolic measures were adjusted for age, sex, and, if applicable, place of recruitment (BWHHS [British Women’s Heart and Health Study] and UKCTOCS [the United Kingdom Collaborative Trial of Ovarian Cancer Screening]) or principal components of genomic ancestry (PEL82 [1982 Pelotas Birth Cohort] and some studies contributing to Metabolomics consortium), and the resulting residuals were transformed to normal distribution by inverse rank-based normal transformation. A indicates the Avon Longitudinal Study of Children and Parents-Mothers’ Cohort; Apo, apolipoprotein; B, BWHHS; C, the Caerphilly Prospective Study; DAG, diglycerides; HDL, high-density lipoprotein; HDL-C, HDL cholesterol; HDLd, HDL particle mean diameter; IDL, intermediate-density lipoprotein; L-HDL, large HDL; L-LDL, large LDL; L-VLDL, large VLDL; LDL, low-density lipoprotein; LDL-C, LDL cholesterol; LDLd, LDL particle mean diameter; M-HDL, medium HDL; M-LDL, medium LDL; M-VLDL, medium VLDL; M, Metabolomics consortium; P, PEL82; S-HDL, small HDL; S-LDL, small LDL; S-VLDL, small VLDL; TG, triglycerides; U, UKCTOCS Nested Case–Control Study; VLDL, very-low-density lipoprotein; VLDL-C, VLDL cholesterol; VLDLd, VLDL particle mean diameter; W, Whitehall II Study; XL-HDL, very large HDL; XL-VLDL, very large VLDL; XS-VLDL, very small VLDL; and XXL-VLDL, extremely large VLDL.

In the multivariable analysis, adiponectin was associated with 59 of 74 (80%) metabolites at nominal level (*P*<0.05) and 49 of 74 (66%) after correcting for multiple testing (*P*<0.0019). Overall, higher circulating adiponectin was associated with a healthier systemic metabolite profile. Blood adiponectin concentration was strongly related to multiple lipoprotein traits. With higher adiponectin concentration, lipid concentration was lower in very low-density lipoprotein (VLDL) subclasses and higher in HDL subclasses, except for small HDL. There was no strong evidence of circulating adiponectin associating with total lipid content in LDL subclasses or in intermediate-density lipoprotein, although adiponectin concentration was inversely associated with LDL-TG. Higher adiponectin was associated with lower concentration of cholesterol, TG, and lower mean particle diameter in VLDL, as well as higher cholesterol concentration and mean particle diameter in HDL. Higher adiponectin concentration was also associated with higher concentration of Apo AI and phospholipids and lower concentration of TG and diglycerides (Figure 2).

**Figure 3.**
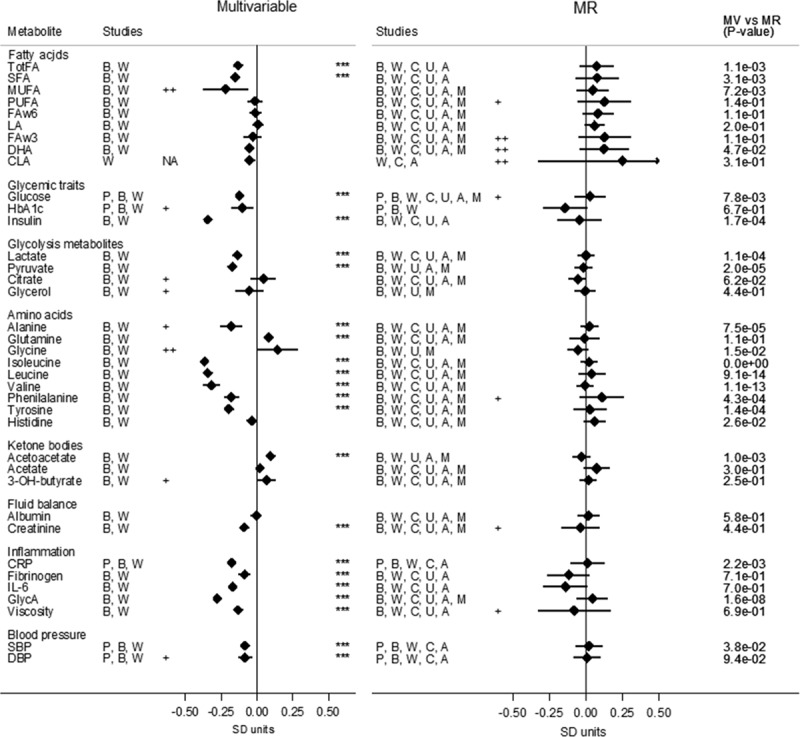
Association of multiple metabolic measures with blood adiponectin levels from multivariable and Mendelian randomization (MR) analysis. Values are expressed as units of standardized metabolite concentration (and 95% CI [confidence interval]) per 1 U increment of standardized log adiponectin levels. *P* values for the association between adiponectin and metabolites are indicated by *** if lower than Bonferroni-adjusted threshold (*P* value <0.0019). Heterogeneity was considered substantial if *I*^2^=50% to 75% (+) or high if *I*^2^>75% (++). *P* values for the comparison between multivariable and MR estimates are displayed in the column MR vs MV (*P* value). Metabolic measures were adjusted for age, sex, and, if applicable, place of recruitment (BWHHS [British Women’s Heart and Health Study] and UKCTOCS [the United Kingdom Collaborative Trial of Ovarian Cancer Screening]) or principal components of genomic ancestry (PEL82 [1982 Pelotas Birth Cohort] and some studies contributing to Metabolomics consortium) and the resulting residuals were transformed to normal distribution by inverse rank-based normal transformation. A indicates the Avon Longitudinal Study of Children and Parents-Mothers’ Cohort; B, BWHHS; C, the Caerphilly Prospective Study; CLA, conjugated linoleic acids; CRP, C-reactive protein; DBP, diastolic blood pressure; DHA, docosahexaenoic acid; FAw3, omega-3 fatty acid; FAw6, omega-6 fatty acid; GlycA, glycoprotein acetyls; HbA1c, glycated hemoglobin; IL-6, interleukin-6; LA, linoleic acid; M, Metabolomics consortium; MUFA, monounsaturated fatty acid; P, PEL82; PUFA, polyunsaturated fatty acids; SBP, systolic blood pressure; SFA, saturated fatty acid; TotFA, total fatty acids; U, UKCTOCS Nested Case–Control Study, and W, Whitehall II Study.

Higher circulating adiponectin was also associated with healthier glycemic status (lower glucose and insulin concentration), lower blood concentration of glycolysis-related metabolites (lactate and pyruvate), saturated fatty acids, systemic inflammatory markers (CRP, fibrinogen, IL-6, glycoprotein acetyls, and blood viscosity), blood pressure, creatinine, and higher ketone bodies (acetoacetate). In addition, higher adiponectin concentration was associated with lower concentrations of free branched-chain amino acids (isoleucine, leucine, and valine), aromatic amino acids (phenylalanine and tyrosine), and alanine and higher concentration of glutamine (Figure 3).

In the multivariable analyses, evidence of heterogeneity in pooled estimates across studies was substantial (*I*^2^=50%–75%) for 12 and high (*I*^2^>75%) for 15 metabolic measures (Figures 2 and 3; Tables IVA and V in the Data Supplement). This did not seem to be accounted by sex (Figures I through IV in the Data Supplement), geographic location (Figures V and VI in the Data Supplement), or high risk of disease (Figures VII and VIII in the Data Supplement). Results were consistent for metabolites not included in the main analysis (Figures IX and X in the Data Supplement).

### Causal Effects of Adiponectin on the Systemic Metabolic Profile

Characteristics of the 4 SNPs (rs6810075, rs16861209, rs17366568, and rs3774261) used in Mendelian randomization and their association with adiponectin concentration are shown in Table 3. Overall, SNPs effect allele frequency was similar across studies. Two SNPs had lower allele frequency in the Metabolomics consortium (rs6810075: 51% versus 65%–69% in other studies; rs16861209: 5% versus 9%–11% in other studies), and 1 SNP had a higher frequency in PEL82 compared with other studies (rs3774261: 49% versus 38%–39% in other studies; Table 3). As expected, the selected SNPs were not associated with classical confounders overall (Table VI in the Data Supplement).

**Table 3.**
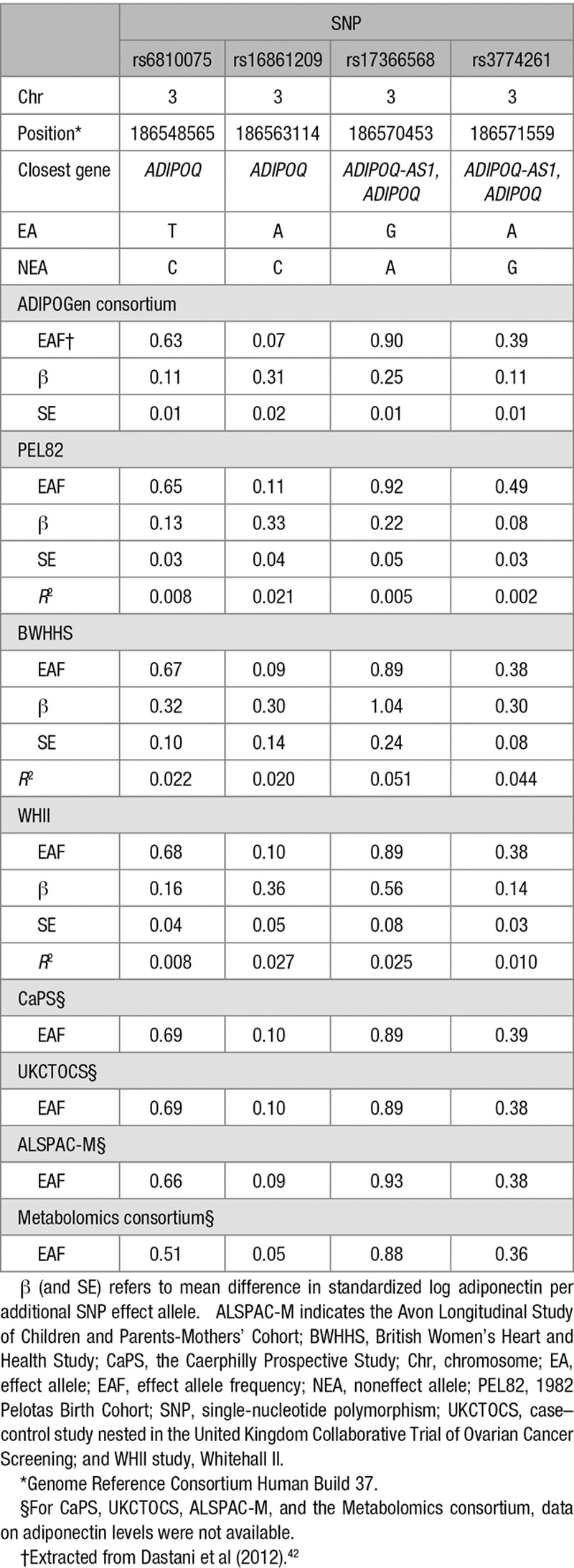
Characteristics of SNPs Selected for Mendelian Randomization Analysis

**Figure 4.**
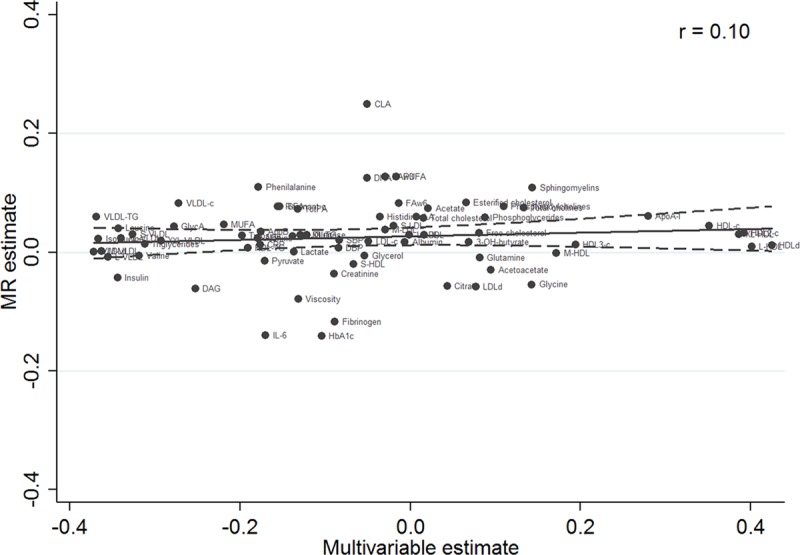
Correlation between estimates from multivariable regression and Mendelian randomization (MR). Apo indicates apolipoprotein; CLA, conjugated linoleic acids, CRP, C-reactive protein; DAG, diglycerides; DBP, diastolic blood pressure; DHA, docosahexaenoic acid; FAw3, omega-3 fatty acid; FAw6, omega-6 fatty acid; GlycA, glycoprotein acetyls; HbA1c, glycated hemoglobin; HDL, high-density lipoprotein; HDL-C, HDL cholesterol; HDLd, HDL particle mean diameter; IDL, intermediate-density lipoprotein; IDL-C, IDL cholesterol; IL-6, interleukin-6; L-HDL, large HDL; L-LDL, large LDL; L-VLDL, large VLDL; LA, linoleic acid; LDL, low-density lipoprotein; LDL-C, LDL cholesterol; LDLd, LDL particle mean diameter M-HDL, medium HDL; M-LDL, medium LDL; M-VLDL, medium VLDL; MUFA, monounsaturated fatty acid; *r*, Pearson correlation coefficient; S-HDL, small HDL; S-LDL, small LDL; S-VLDL, small VLDL; SBP, systolic blood pressure; SFA, saturated fatty acid; TG, triglycerides; VLDL, very-low-density lipoprotein; VLDL-C, VLDL cholesterol; VLDLd, VLDL particle mean diameter; XL-HDL, very large HDL; XL-VLDL, very large VLDL; XS-VLDL, very small VLDL; and XXL-VLDL, extremely large VLDL.

Findings from Mendelian randomization analysis were largely inconsistent with results from multivariable analysis. First, there was no evidence that adiponectin influenced HDL and VLDL traits (Figure 2). Second, genetically increased adiponectin levels were not associated with glycemic traits, free amino acids, and glycolysis-related metabolites (Figure 3). Results were less conclusive for some inflammatory markers (IL-6 and fibrinogen; Figure 3). Third, there was strong statistical evidence that associations from multivariable and Mendelian randomization analyses were inconsistent with each other (Figures 2 and 3), and the overall correlation between multivariable and Mendelian randomization estimates was low (*r*=0.10; Figure 4). Finally, in the Mendelian randomization analysis, adiponectin was not associated with any of the metabolic measures at either *P*<0.05 or *P*<0.0019.

In the Mendelian randomization analyses, evidence of heterogeneity in pooled estimates across studies was substantial (*I*^2^=50%–75%) for 14 and high (*I*^2^>75%) for 3 metabolic measures, suggesting lower heterogeneity in models from genetic analysis than from the multivariable analyses (Figures 2 and 3; Tables IVB and V in the Data Supplement). This did not seem to be driven by sex differences (Figures I through IV in the Data Supplement), geographic location/ethnicity (Figures V and VI in the Data Supplement), or high risk of disease (Figures VII and VIII in the Data Supplement). Results were consistent with no association between adiponectin and metabolites not included in the main analysis (Figures IX and X in the Data Supplement).

## Discussion

In ≤5909 adults, we found using multivariable regression analyses that circulating adiponectin was associated with a pattern of systemic metabolites levels associated with good health. Higher blood adiponectin concentration was associated with higher HDL lipids and lower VLDL lipids, glycemia, and branched-chain amino acids levels. However, when we used genetic variants in the vicinity of adiponectin-encoding gene to test the causal effect of adiponectin on systemic metabolic profiles among ≤37 545 adults, we found little evidence that the associations were causal.

Genetic association studies indicate that genetic variants associated with circulating adiponectin (in loci other than *ADIPOQ*) are also associated with cardiometabolic outcomes, such as type 2 diabetes mellitus^42^ and coronary heart disease^41^; however, this is likely to be reflecting a pleiotropic effect of these variants. Our findings and previous Mendelian randomization studies^19,22^ suggest that the association between circulating adiponectin and metabolic biomarkers and cardiometabolic diseases is likely to be explained by shared factors (confounding) rather than by a direct role of adiponectin on metabolism and downstream cardiometabolic disease. These results are in contrast to findings from animal models pointing to insulin-sensitizing and antiatherogenic actions of adiponectin.^1^

Circulating adiponectin is known to be substantially reduced among obese individuals, particularly in the presence of central fat accumulation.^45^ A recent Mendelian randomization study examining the causal metabolic effects of body mass index demonstrated that lower body mass index was related to favorable lipoprotein subclass profile and lower concentration of branched-chain amino acids, inflammatory markers, and insulin,^34^ which is remarkably similar to our results from the conventional multivariable analysis. In addition, numerous studies have shown that adiponectin production is suppressed by insulin action in humans, which seems to be at least partly attributed to regulation at the transcriptional level.^46^ As an example, elevated circulating adiponectin is found in contexts of both primary deficiency of insulin (type 1 diabetes mellitus)^47^ and global insulin resistance because of genetic or acquired defects in the insulin receptor.^48^ Genetic predisposition to insulin resistance and central fat accumulation^45,49^ is related to lower circulating adiponectin. Evidence from animal models has raised the possibility of a bidirectional relationship between adiponectin and insulin concentration.^50^ Early Mendelian randomization studies did indicate that adiponectin could mitigate insulin resistance^20,21^; however, these results could not be replicated in a larger Mendelian randomization study,^19^ as well as in our study presented here. The well-known metabolic effects of adiposity and insulin on circulating adiponectin concentration reinforce that the clustering of adiponectin and several traditional and novel biomarkers is likely to result from confounding because of increasing adiposity and disruption of insulin action.

Strengths of our study include detailed metabolic profile in several longitudinal studies, which enabled us to characterize the metabolic profile of high adiponectin concentration beyond traditional biomarkers, as well as the use of Mendelian randomization to disentangle the causal effect of adiponectin on the metabolism. Mendelian randomization analysis can reliably test for the presence of a causal relation under the 3 assumptions of an instrumental variable that the genetic variants (1) are robustly associated with the risk factor of interest (adiponectin), (2) should only affect the outcome (metabolites) through the exposure, and (3) are not associated with exposure–outcome confounders.^51^ To ensure that the instrumental variable assumptions were met, or were at least plausible, we only used SNPs strongly and specifically (within *ADIPO*Q gene) related to adiponectin concentration as instrumental variables and we adjusted for population structure in models using data from PEL82 to avoid confounding by population stratification. One of the limitations of our study was the limited power in subgroup analyses including only individual-level data (sex- and risk-stratified analyses), which limited our investigation of potential sources of heterogeneity. Another limitation was the absence of data on high–molecular weight adiponectin, which is believed to account for most of the adiponectin biological effects in experimental settings. However, most human (and many animal model) studies have not used high–molecular weight adiponectin, and we found the same multivariable observational associations as in previous studies. Also, it should be emphasized that SNPs in *ADIPOQ* gene are associated with both total and high–molecular weight adiponectin,^52–54^ including SNPs we used in our analysis (eg, rs3774261)^52^ or others in high linkage disequilibrium with these (eg, rs16861209 is highly correlated with rs17300539 − *R*^2^ >0.8).^53,54^

Overall, our findings suggest that low circulating adiponectin is likely to reflect adipocyte dysfunction and that altered total blood adiponectin concentration is an epiphenomenon in the context of metabolic disease, rather than a key determinant. Therefore, interventions targeting manipulation of adiponectin concentration are unlikely to result in therapeutic benefits for tackling cardiometabolic diseases. Our results highlight the potential of Mendelian randomization analysis and high-throughput metabolomics profiling to yield important insights to advance our understanding in the pathophysiology of common complex diseases and to inform which targets are best bets for taking forward into drug development, given that drug target validation is a key obstacle underlying the unsustainably high rate of drug development failure. Although our and other studies suggest that adiponectin is not a valuable target for developing drugs aimed at preventing cardiometabolic diseases, it may nonetheless be a valuable biomarker for predicting these diseases given the wide-ranging associations shown here. The associations we have found would need to be replicated in additional independent studies before testing their ability to predict disease outcomes.

## Acknowledgments

We acknowledge Andy Ryan for his contribution to data collection from UKCTOCS (the United Kingdom Collaborative Trial of Ovarian Cancer Screening). Summary genome-wide association data on adiponectin have been contributed by ADIPOGen Consortium and have been downloaded from https://www.mcgill.ca/genepi/adipogen-consortium. Summary genome-wide association data on metabolic measures have been contributed by Kettunen et al^31^ and have been downloaded from http://www.computationalmedicine.fi/data#NMR_GWAS.

## Sources of Funding

Drs Borges, Ferreira, Lawlor, and Gaunt work in the MRC Integrative Epidemiology Unit at the University of Bristol that receives funding from the UK Medical Research Council (MC_UU_12013/5 and MC_UU_12013/8). Dr Borges is supported by MRC Skills Development Fellowship (MR/P014054/1). Dr Lawlor is a UK National Institute of Health Research Senior Investigator (NF-SI-0611-10196). Dr Kivimaki is supported by the UK Medical Research Council (K013351). PEL82 (the 1982 Pelotas Birth Cohort) is conducted by Postgraduate Program in Epidemiology at Universidade Federal de Pelotas with the collaboration of the Brazilian Public Health Association (ABRASCO). From 2004 to 2013, the Wellcome Trust supported PEL82. The International Development Research Center, World Health Organization, Overseas Development Administration, European Union, National Support Program for Centers of Excellence (PRONEX), the Brazilian National Research Council (CNPq), and the Brazilian Ministry of Health supported previous phases of the study. The UCL-LSHTM-Edinburgh-Bristol (UCLEB) consortium, which is supported by the British Heart Foundation Program Grant RG/10/12/28456, consists of 12 studies: NPHS II (Northwick Park Heart Study II), BRHS (British Regional Heart Study), WHII study (Whitehall II), ELSA (English Longitudinal Study of Ageing), MRC NSHD (Medical Research Council National Survey of Health and Development), 1958BC (1958 Birth cohort), CaPS (Caerphilly Prospective Study), BWHHS (British Women’s Heart and Health Study), EAS (Edinburgh Artery Study), EHDPS (Edinburgh Heart Disease Prevention Study), ET2DS (Edinburgh Type 2 Diabetes Study), and AAAT (Asymptomatic Atherosclerosis Aspirin Trial). BWHHS is supported by funding from the British Heart Foundation and the Department of Health Policy Research Programme (England). EAS is funded by the British Heart Foundation (Programme Grant RG/98002), with Metabochip genotyping funded by a project grant from the Chief Scientist Office of Scotland (Project Grant CZB/4/672). The WHII study is supported by grants from the Medical Research Council (K013351), British Heart Foundation (RG/07/008/23674), Stroke Association, the US National Heart Lung and Blood Institute (5RO1 HL036310), the US National Institute on Aging (5RO1AG13196), the US Agency for Healthcare Research and Quality (HS06516), and the John D. and Catherine T. MacArthur Foundation Research Networks on Successful Midlife Development and Socio-economic Status and Health. CaPS was funded by the Medical Research Council and undertaken by the former MRC Epidemiology Unit (South Wales). The CaPS DNA bank was established with funding from an MRC project grant. The CaPS data archive is maintained by the University of Bristol. MRC Integrative Epidemiology Unit, Bristol, is supported by MRC grants (MR_UU_12013/1, MR_UU_12013/5 and MR_UU_12013/8). UKCTOCS (the United Kingdom Collaborative Trial of Ovarian Cancer Screening) was funded by the Medical Research Council (G9901012 and G0801228), Cancer Research UK (C1479/A2884), and the Department of Health, with additional support from The Eve Appeal. Phenotypic data for this case–control data set were supported by the National Institute for Health Research, Biomedical Research Centre at University College London Hospital. ALSPAC-M (Cohort of Mothers From the Avon Longitudinal Study of Children and Parents) phenotypic data were collected with funding from the British Heart Foundation (SP/07/008/24066), Wellcome Trust (WT092830M), and UK Research Councils (UKRC) via the MRC (G1001357); genetic data collection was funded by the Wellcome Trust (WT088806). In addition, the ALSPAC full study receives core support from the University of Bristol, UK Medical Research Council and the Wellcome Trust (102215/2/13/2) and the University of Bristol. The ALSPAC team is extremely grateful to all the families who took part in this study, the midwives for their help in recruiting them, and the whole ALSPAC team, which includes interviewers, computer and laboratory technicians, clerical workers, research scientists, volunteers, managers, receptionists, and nurses.

## Disclosures

Dr Menon has stock ownership in and research funding from Abcodia Pvt Ltd. The other authors report no conflicts.

## Supplementary Material

## CLINICAL PERSPECTIVE

Adiponectin, a protein produced by adipose cells, has insulin-sensitizing, anti-inflammatory, antiatherogenic, and cardiomyocyte-protective properties in animal models. In prospective studies in humans, higher circulating adiponectin is associated with lower risk of type 2 diabetes mellitus, hepatic dysfunction, and metabolic syndrome. However, it is not clear whether adiponectin is protective against these metabolic disorders or whether these associations are just reflecting reverse causality (where disease status could alter adiponectin concentration) or residual confounding (where adiponectin could be a marker of another causal factor, such as adiposity or insulin resistance). We used Mendelian randomization to clarify whether circulating adiponectin is causally related to the metabolic profile of ≤37 545 adults. Four common genetic variants nearby the gene encoding adiponectin (*ADIPOQ*) were used as instruments to test the effect of circulating adiponectin on 74 metabolic measures selected to broadly represent the systemic metabolite profile, including lipoprotein subclasses, fatty acids, glycemic traits, free amino acids, inflammatory markers, and blood pressure. Overall, our findings do not support a direct role of circulating adiponectin on the systemic metabolic profile in humans and indicate that the clustering of adiponectin and several traditional and novel biomarkers is likely to result from confounding or reverse causality. Therefore, interventions targeting manipulation of adiponectin concentration are unlikely to result in therapeutic benefits for tackling metabolic diseases.
