# Supplementary material for: Metabolic Profiling of Adiponectin Levels in Adults: Mendelian Randomization Analysis
Source: Circ Cardiovasc Genet. 2017 Dec 13;10(6):e001837. doi: 10.1161/CIRCGENETICS.117.001837 (PMC5736126; doi:10.1161/CIRCGENETICS.117.001837)
Supplement: Supplementary file 1 [file hcg-10-e001837-s001.pdf]

SUPPLEMENTAL MATERIAL

SUPPLEMENTARY METHODS

Nuclear magnetic resonance (NMR) spectroscopy platform

Over 150 quantified metabolomic measures were obtained per sample of EDTA-plasma, using a 1D proton (^1H) NMR spectroscopy-based platform described previously (1-4). Briefly, the serum samples were stored in a freezer at -80ºC. The frozen samples were first slowly thawed in a refrigerator (+4ºC) overnight prior to metabolomics profiling. 260 µL plasma and 260 µL sodium phosphate buffer (75 mM Na2HPO4, 0.08% sodium 3-(trimethylsilyl)propionate-2,2,3,3-d4, 0.04% sodium azide in 80%/20% H20/D2O, pH 7.4) were mixed and transferred to NMR tubes using an 8-channel, Varispan Janus liquid handling robot (PerkinElmer). NMR spectra were acquired using a Bruker Avance III HD 500MHz spectrometer with a room temperature 5mm, inverse triple resonance TXI probe and a Bruker Avance III HD 600MHz spectrometer equipped with a nitrogen-cooled triple resonance probe (CryoProbe Prodigy TCI). Both spectrometers were equipped with SampleJet auto-samplers with cooled (6°C) sample storage. Spectra were acquired using standardized parameters using three NMR experiments or 'molecular windows' to characterize lipoproteins, low molecular weight metabolites and lipids. Lipid spectra were acquired after a standardised lipid extraction procedure performed on each sample using a VIAFLO 96 channel electronic pipette (Integra Biosciences). Data pre-processing and quantification were as previously described (1-4). The NMR spectra were analysed for absolute quantification using regression models (5). The 14 lipoprotein subclass sizes were defined as follows: very low density lipoprotein (VLDL) is subdivided into six subclasses, the largest being extremely large VLDL with particle diameters from 75 nm upwards and a possible contribution of chylomicrons, and five remaining VLDL subclasses (average particle diameters of 64.0 nm, 53.6 nm, 44.5 nm, 36.8 nm, and 31.3 nm); Intermediate density lipoprotein (IDL) (28.6 nm), three low density lipoprotein (LDL) subclasses (25.5 nm, 23.0 nm, and 18.7 nm), and four high density lipoprotein (HDL) subclasses (14.3 nm, 12.1 nm, 10.9 nm, and 8.7 nm). The mean sizes for VLDL, LDL and HDL particles were calculated by weighting the corresponding subclass diameters with their particle concentrations (4). There is a high analytical consistency, in epidemiological settings, between metabolic measures quantified by the NMR metabolomics platform and the concentrations obtained from routine clinical chemistry (6), and other analytical methods, such as gas chromatography (6, 7) and enzymatic

method (6), with correlations >0.9. In addition, the consistency of biomarker associations with disease incidence for metabolic traits quantified by NMR and two widely used mass spectroscopy platforms has been demonstrated (6, 7).

Selection of genetic variants

The SNPs used for the Mendelian randomization analysis were selected from 145 SNPs with good evidence ($p < 5*10^{-8}$) for association with blood adiponectin concentration in the European ancestry GWAS meta-analysis from the ADIPOGen consortium (8). ADIPOGen participating studies tested for the additive genetic association of SNPs with natural log transformed adiponectin levels, while adjusting for age, sex, BMI, principal components of population stratification and study site (where appropriate), and for family structure in cohorts with family members. Independent SNPs within the *ADIPOQ* locus (± 50 kb) have been previously selected by Dastani et al (2013) (9) by linkage disequilibrium (LD) prunning of the genome-wide significant SNPs, retaining SNPs that explained most variance in adiponectin concentration in each LD block (LD threshold: $R^2 < 0.05$ in HapMap CEU population (Utah residents with Northern and Western European ancestry)).

The discovery sample from ADIPOGen GWAS was largely independent from the sample used to estimate the association between SNP and metabolites in our study. We estimated that, depending on the metabolite, between zero and 26% of participants included in the analysis of SNP-metabolites association would have been included in the discovery ADIPOGen GWAS.

Mendelian randomization analyses

The two-sample Mendelian randomization estimates and respective standard errors were obtained by meta-analyzing SNP-specific Wald ratios (i.e. ratio between SNP-outcome and SNP-exposure association) with the following formulas:

$$\hat{\beta} = \frac{\sum_{k=1}^{K} X_k Y_k \, \sigma_{yk}^{-2}}{\sum_{k=1}^{K} X_k^2 \sigma_{yk}^{-2}} \qquad SE_{\hat{\beta}} = \sqrt{\frac{1}{\sum_{k=1}^{K} X_k^2 \sigma_{yk}^{-2}}}$$

Where X_k is the mean change in standardized log adiponectin units per additional effect allele of SNP k and Y_k is the mean change in standardized units of metabolic measures per additional effect allele of SNP k with standard error σ_{Yk}. To increase precision and avoid bias due to statistical overfitting, estimates for X_k were obtained from ADIPOGen consortium dataset (8). Prior to analysis, estimates from ADIPOGen consortium were standardized (converted from log adiponectin to standardized log adiponectin units) using individual level data from PEL82 with a similar adiponectin distribution (adiponectin concentration in ADIPOGen consortium: mean = 9.8 µg/ml (standard deviation = 5.6); adiponectin concentration in 1982 Pelotas Birth Cohort: mean = 9.3 µg/ml (standard deviation = 5.7)). Estimates for Y_k were derived from each study using linear regression models considering an additive model for SNP alleles.

Comparison between multivariable and Mendelian randomization analyses

Results from conventional multivariable and Mendelian randomization analyses were compared using the Z-test:

$$Z = (\beta_{mva} - \beta_{MR})/\sqrt(SE_{mva}^2 + SE_{MR}^2)$$

Where β_{mv} represents estimates from conventional multivariable analysis (with respective standard error, SE_{mv}) and β_{MR} represents estimates from Mendelian randomization analysis (with respective standard error, SE_{MR}).

Proportion of variance in adiponectin concentration explained by genetic instruments

In order to estimate the strength of our genetic instruments, we estimated the phenotypic variance explained by a given SNP (R^2) for adiponectin concentration. We used ADIPOGen summary data to approximate R^2 for a given SNP based on the effect estimate for its association with the trait of interest (beta or $\hat{\beta}$), respective standard error ($se(\hat{\beta})$), minor allele frequency (MAF), and sample size (N). The following formula was used as previously described by Shim et al., 2015 (10):

$$R^2 \cong \frac{2\hat{\beta}^2 MAF(1-MAF)}{2\hat{\beta}^2 MAF(1-MAF) + (se(\hat{\beta}))^2 2NMAF(1-MAF)}$$

The phenotypic variance explained by the composite genetic instrument (combining all SNPs) was estimated by the sum of SNP-specific R^2 as shown below:

SNPs used as instrumental variables for adiponectin concentration in Mendelian randomization analysis and association with adiponectin concentration

rs ID	Chr	EA	NEA	EAF	R^2	N
rs6810075	3	T	C	0.63	0.0066	29140
rs16861209	3	A	C	0.01	0.0125	29199
rs17366568	3	G	A	0.91	0.0125	24865
rs3774261	3	A	G	0.60	0.0080	29081
Combined instrument	N/A	N/A	N/A	N/A	0.0396	N/A

Chr: chromosome; EA: effect allele (trait-increasing allele); NEA: non-effect allele; R^2: proportion of phenotypic variance explained by SNP; Beta: increase in standardized log adiponectin concentration per EA; SE: standard error; N: sample size; N/A: not applicable.

References

1. Soininen P, Kangas AJ, Wurtz P, Tukiainen T, Tynkkynen T, Laatikainen R, et al. High-throughput serum NMR metabonomics for cost-effective holistic studies on systemic metabolism. Analyst. 2009;134(9):1781-5.
2. Inouye M, Kettunen J, Soininen P, Silander K, Ripatti S, Kumpula LS, et al. Metabonomic, transcriptomic, and genomic variation of a population cohort. Mol Syst Biol. 2010;6:441.
3. Soininen P, Kangas AJ, Wurtz P, Suna T, Ala-Korpela M. Quantitative serum nuclear magnetic resonance metabolomics in cardiovascular epidemiology and genetics. Circ Cardiovasc Genet. 2015;8(1):192-206.
4. Kujala UM, Makinen VP, Heinonen I, Soininen P, Kangas AJ, Leskinen TH, et al. Long-term leisure-time physical activity and serum metabolome. Circulation. 2013;127(3):340-8.
5. Vehtari A, Makinen VP, Soininen P, Ingman P, Makela SM, Savolainen MJ, et al. A novel Bayesian approach to quantify clinical variables and to determine their spectroscopic counterparts in 1H NMR metabonomic data. BMC Bioinformatics. 2007;8 Suppl 2:S8.
6. Würtz P, Kangas A, Soininen P, Lawlor D, Davey Smith G, Ala-Korpela M. Quantitative serum NMR metabolomics in large-scale epidemiology: a primer on -omic technology. American Journal of Epidemiology. 2017.
7. Wurtz P, Havulinna AS, Soininen P, Tynkkynen T, Prieto-Merino D, Tillin T, et al. Metabolite profiling and cardiovascular event risk: a prospective study of 3 population-based cohorts. Circulation. 2015;131(9):774-85.

8. Dastani Z, Hivert MF, Timpson N, Perry JR, Yuan X, Scott RA, et al. Novel loci for adiponectin levels and their influence on type 2 diabetes and metabolic traits: a multi-ethnic meta-analysis of 45,891 individuals. PLoS Genet. 2012;8(3):e1002607.
9. Dastani Z, Johnson T, Kronenberg F, Nelson CP, Assimes TL, März W, et al. The shared allelic architecture of adiponectin levels and coronary artery disease. Atherosclerosis. 2013;229(1):145-8.
10. Shim H, Chasman DI, Smith JD, Mora S, Ridker PM, Nickerson DA, et al. A multivariate genome-wide association analysis of 10 LDL subfractions, and their response to statin treatment, in 1868 Caucasians. PLoS One. 2015;10(4):e0120758.

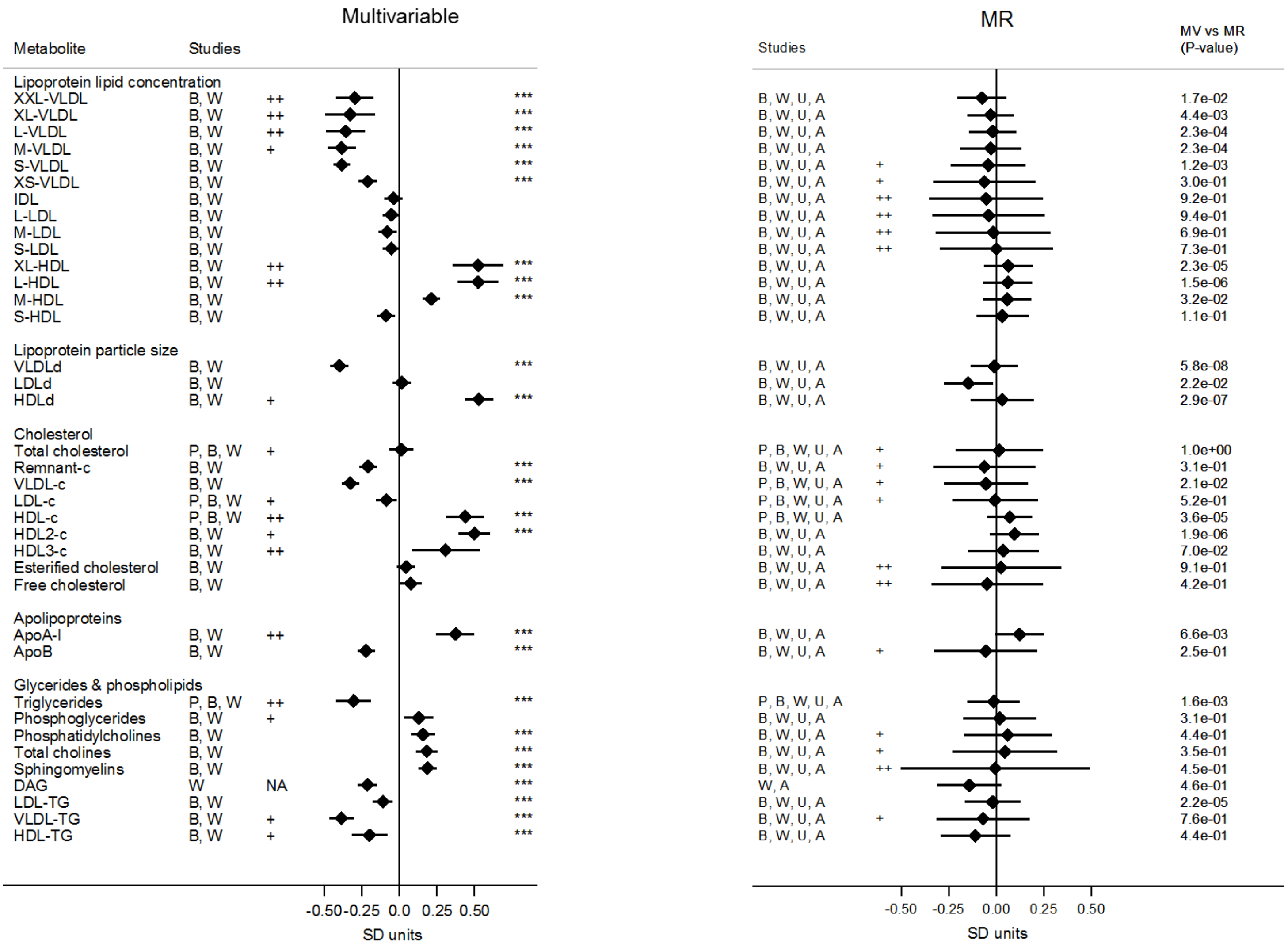

Supplementary figure 1. Association of lipoprotein traits with blood adiponectin levels from observational and Mendelian randomization (MR) analysis among women.

Values are expressed as units of standardized log metabolite concentration (and 95% CI) per 1 unit increment of standardized log adiponectin levels. P-values for the association between adiponectin and metabolites are indicated by three asterisks ("***") if lower than Bonferroni-adjusted threshold (P-value < 0.0019). Heterogeneity was considered substantial if I^2 = 50-75% ("+") or very high if I^2 > 75% ("++"). P-values for the comparison between multivariable and Mendelian randomization estimates are displayed in the column "MR vs MV (P-value)". Metabolic measures were adjusted for age, sex, and, if applicable, place of recruitment (BWHHS and UKCTOCS) or principal components of genomic ancestry (PEL82 and some studies contributing to Metabolomics consortium) and the resulting residuals were transformed to normal distribution by inverse rank-based normal transformation. XXL: extremely large, XL: very large, L: large, M: medium, S: small, XS: very small, VLDL: very low-density lipoprotein, LDL: low-density lipoprotein, IDL: intermediate-density lipoprotein, HDL: high-density lipoprotein, c: cholesterol, DAG: diglycerides, TG: triglycerides, P: 1982 Pelotas Birth Cohort, B: British Women Heart and Health Study, W: Whitehall II Study, U: UKCTOCS nested case-control study, , A: The Avon Longitudinal Study of Children and Parents – mothers' cohort, SD units: standard deviation units, CI: confidence interval.

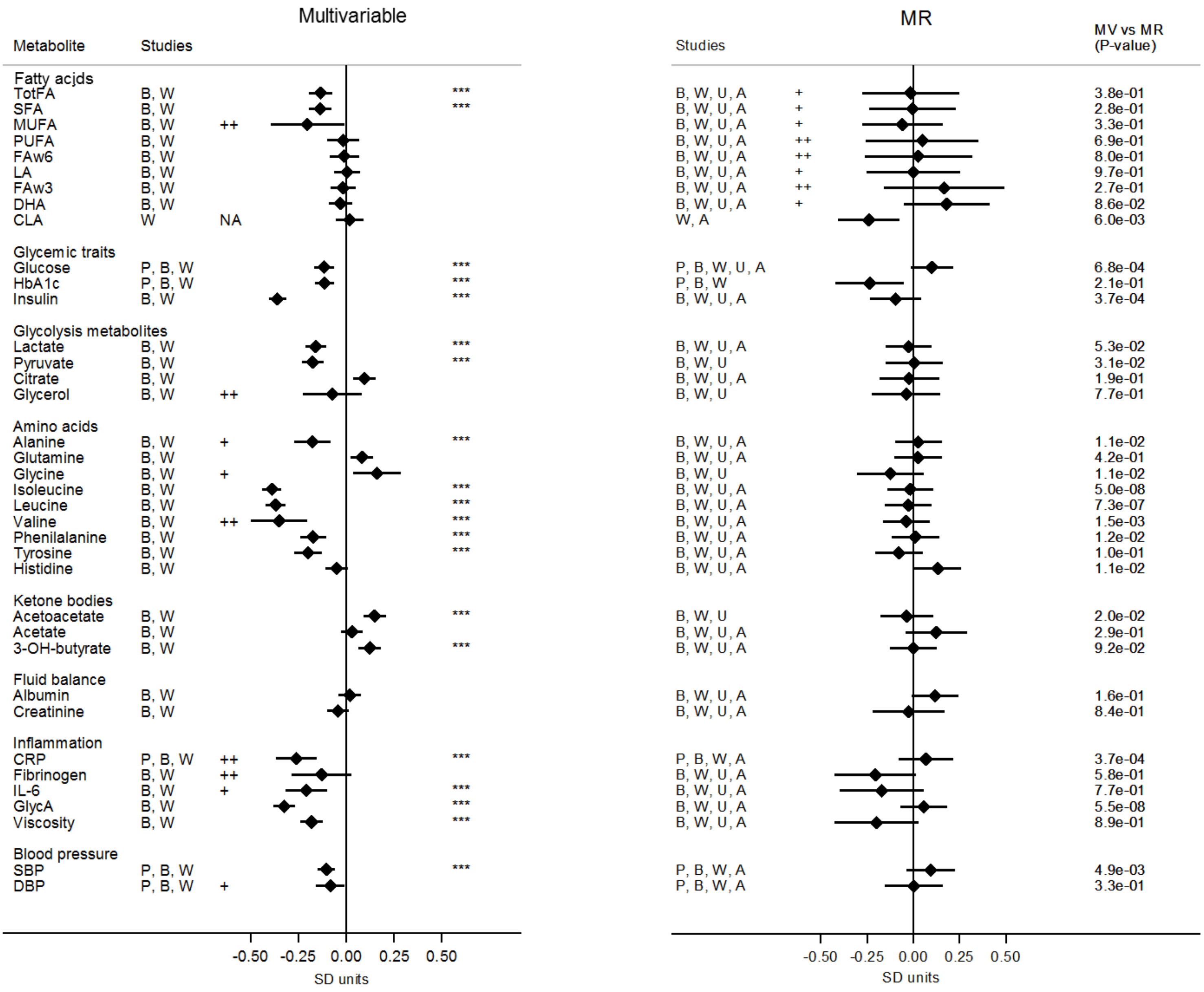

Supplementary figure 2. Association of multiple metabolic measures with blood adiponectin levels from observational and Mendelian randomization analysis among women.

Values are expressed as units of standardized log metabolite concentration (and 95% CI) per 1 unit increment of standardized log adiponectin levels. P-values for the association between adiponectin and metabolites are indicated by three asterisks ("***") if lower than Bonferroni-adjusted threshold (P-value < 0.0019). Heterogeneity was considered substantial if I^2 = 50-75% ("+") or very high if I^2 > 75% ("++"). P-values for the comparison between multivariable and Mendelian randomization estimates are displayed in the column "MR vs MV (P-value)". Metabolic measures were adjusted for age, sex, and, if applicable, place of recruitment (BWHHS and UKCTOCS) or principal components of genomic ancestry (PEL82 and some studies contributing to Metabolomics consortium) and the resulting residuals were transformed to normal distribution by inverse rank-based normal transformation. TotFA: total fatty acids, SFA: saturated fatty acid, MUFA: monounsaturated fatty acid, PUFA: polyunsaturated fatty acids, FAw6: omega-6 fatty acid, LA: linoleic acid, FAw3: omega-3 fatty acid, DHA: docosaexaenoic acid, CLA: conjugated linoleic acids, HbA1c: glycated haemoglobin, CRP: c-reactive protein, IL-6: interleukin-6, GlycA: glycoprotein acetyls, SBP: systolic blood pressure, DBP: diastolic blood pressure, P: 1982 Pelotas Birth Cohort, B: British Women Heart and Health Study, W: Whitehall II Study, U: UKCTOCS nested case-control study, A: The Avon Longitudinal Study of Children and Parents – mothers' cohort, SD units: standard deviation units, CI: confidence interval.

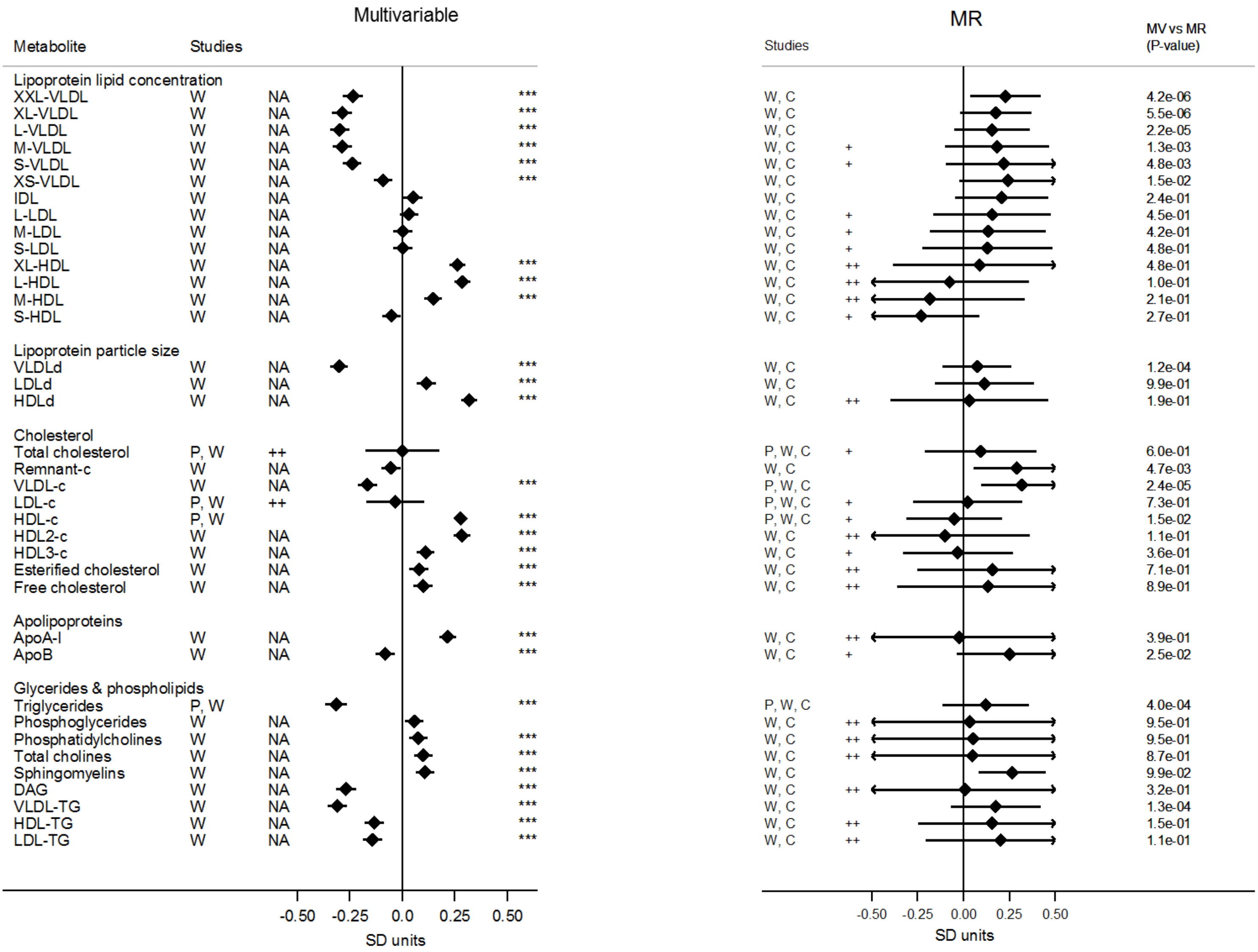

Supplementary figure 3. Association of lipoprotein traits with blood adiponectin levels from observational and Mendelian randomization (MR) analysis among men.

Values are expressed as units of standardized log metabolite concentration (and 95% CI) per 1 unit increment of standardized log adiponectin levels. P-values for the association between adiponectin and metabolites are indicated by three asterisks ("***") if lower than Bonferroni-adjusted threshold (P-value < 0.0019). Heterogeneity was considered substantial if I^2 = 50-75% ("+"), very high if I^2 > 75% ("++") or not applicable ("NA") when only one study contributed to the estimate. P-values for the comparison between multivariable and Mendelian randomization estimates are displayed in the column "MR vs MV (P-value)". Metabolic measures were adjusted for age, sex, and, if applicable, place of recruitment (BWHHS and UKCTOCS) or principal components of genomic ancestry (PEL82 and some studies contributing to Metabolomics consortium) and the resulting residuals were transformed to normal distribution by inverse rank-based normal transformation. XXL: extremely large, XL: very large, L: large, M: medium, S: small, XS: very small, VLDL: very low-density lipoprotein, LDL: low-density lipoprotein, IDL: intermediate-density lipoprotein, HDL: high-density lipoprotein, c: cholesterol, DAG: diglycerides, TG: triglycerides, P: 1982 Pelotas Birth Cohort, W: Whitehall II Study, C: The Caerphilly Prospective Study, SD units: standard deviation units, CI: confidence interval.

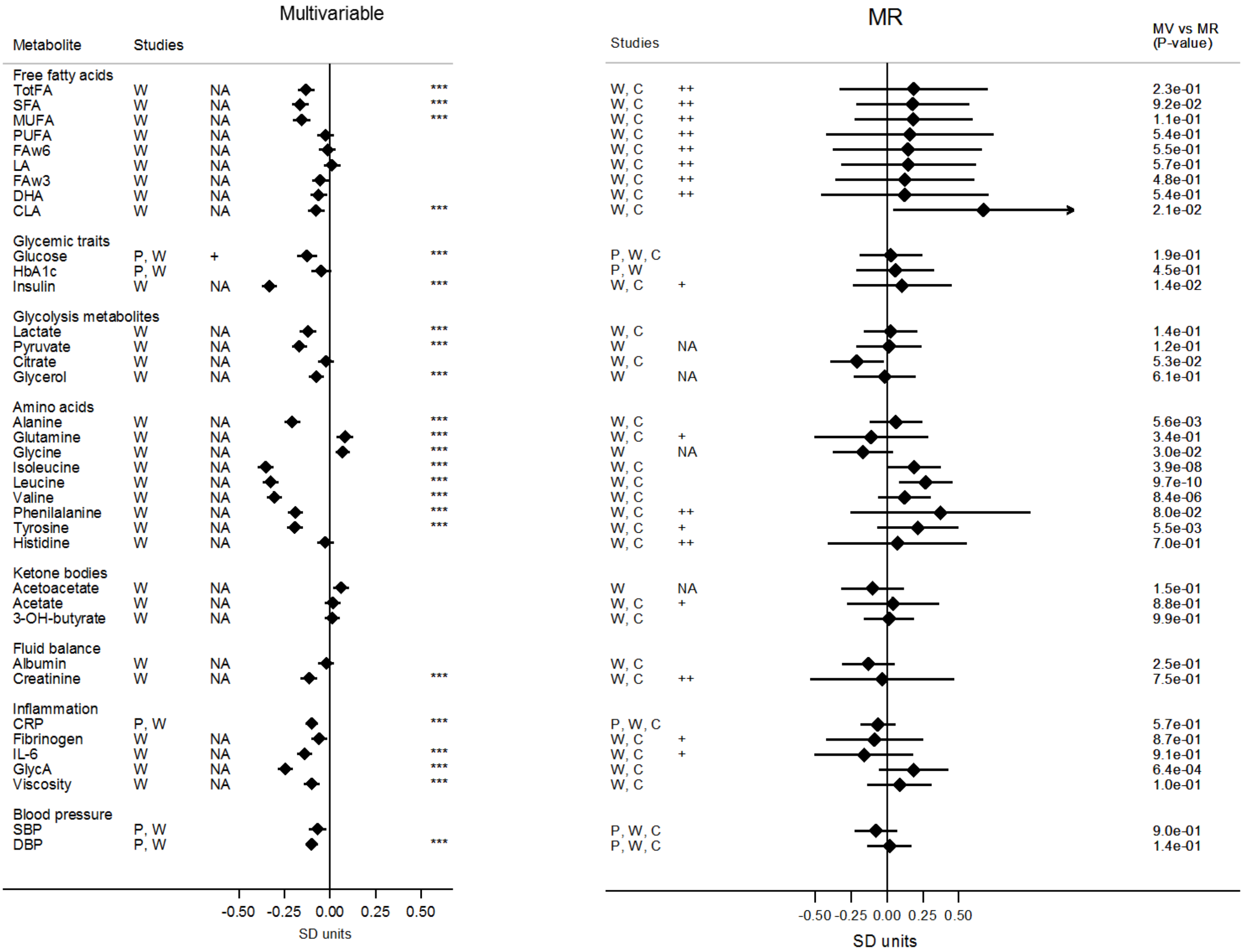

Supplementary figure 4. Association of multiple metabolic measures with blood adiponectin levels from observational and Mendelian randomization analysis among men.

Values are expressed as units of standardized log metabolite concentration (and 95% CI) per 1 unit increment of standardized log adiponectin levels. P-values for the association between adiponectin and metabolites are indicated by three asterisks ("***") if lower than Bonferroni-adjusted threshold (P-value < 0.0019). Heterogeneity was considered substantial if I^2 = 50-75% ("+") or very high if I^2 > 75% ("++") or not applicable ("NA") when only one study contributed to the estimate. P-values for the comparison between multivariable and Mendelian randomization estimates are displayed in the column "MR vs MV (P-value)". Metabolic measures were adjusted for age, sex, and, if applicable, place of recruitment (BWHHS and UKCTOCS) or principal components of genomic ancestry (PEL82 and some studies contributing to Metabolomics consortium) and the resulting residuals were transformed to normal distribution by inverse rank-based normal transformation. TotFA: total fatty acids, SFA: saturated fatty acid, MUFA: monounsaturated fatty acid, PUFA: polyunsaturated fatty acids, FAw6: omega-6 fatty acid, LA: linoleic acid, FAw3: omega-3 fatty acid, DHA: docosaexaenoic acid, CLA: conjugated linoleic acids, HbA1c: glycated haemoglobin, CRP: c-reactive protein, IL-6: interleukin-6, GlycA: glycoprotein acetyls, SBP: systolic blood pressure, DBP: diastolic blood pressure, P: 1982 Pelotas Birth Cohort, W: Whitehall II Study, C: The Caerphilly Prospective Study, SD units: standard deviation units, CI: confidence interval.

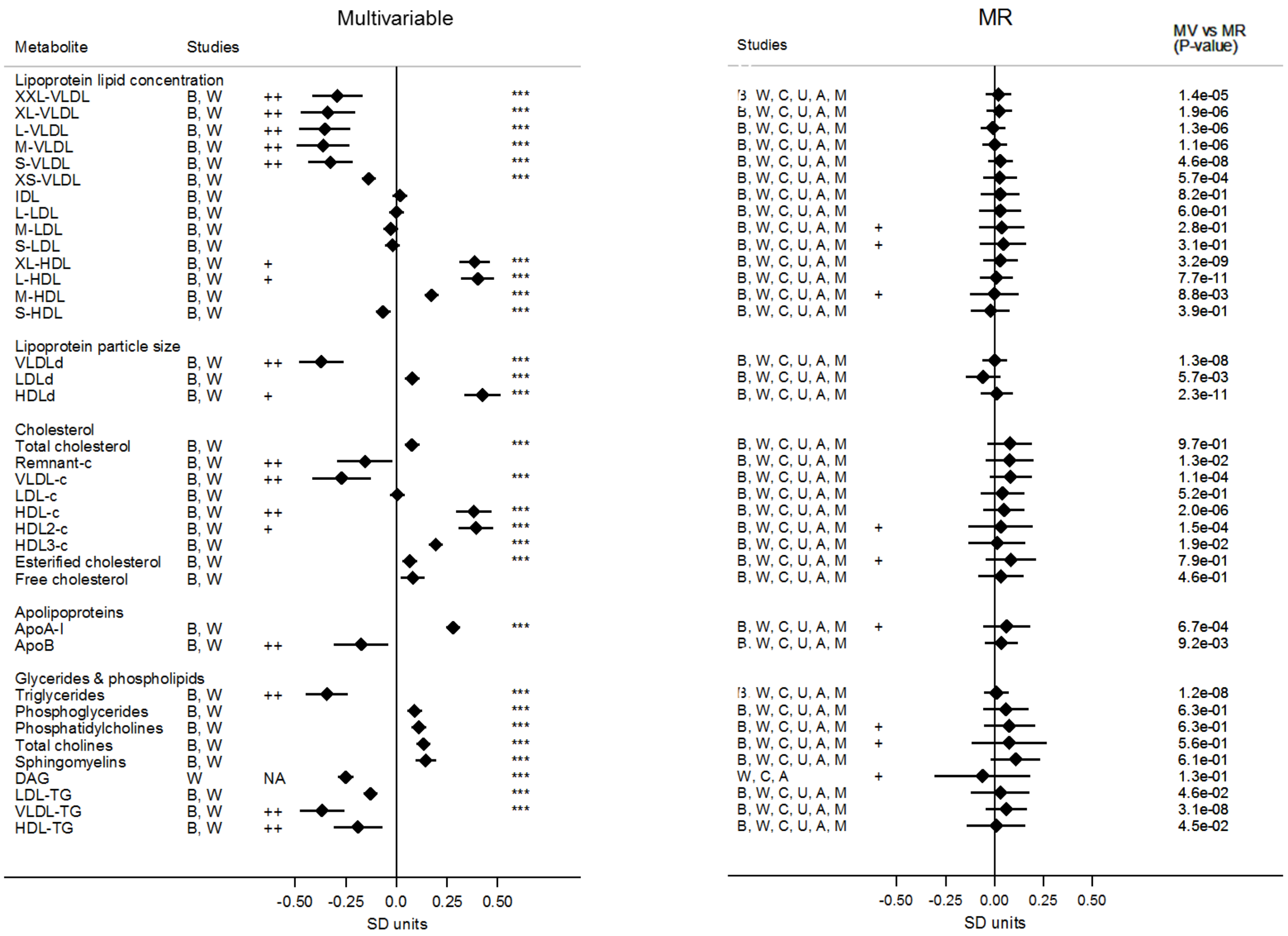

Supplementary figure 5. Association of lipoprotein traits with blood adiponectin levels from observational and Mendelian randomization (MR) analysis restricted to individuals of European ancestry.

Values are expressed as units of standardized log metabolite concentration (and 95% CI) per 1 unit increment of standardized log adiponectin levels. P-values for the association between adiponectin and metabolites are indicated by three asterisks ("***") if lower than Bonferroni-adjusted threshold (P-value < 0.0019). Heterogeneity was considered substantial if I^2 = 50-75% ("+") or very high if I^2 > 75% ("++"). P-values for the comparison between multivariable and Mendelian randomization estimates are displayed in the column "MR vs MV (P-value)". Metabolic measures were adjusted for age, sex, and, if applicable, place of recruitment (BWHHS and UKCTOCS) or principal components of genomic ancestry (PEL82 and some studies contributing to Metabolomics consortium) and the resulting residuals were transformed to normal distribution by inverse rank-based normal transformation. XXL: extremely large, XL: very large, L: large, M: medium, S: small, XS: very small, VLDL: very low-density lipoprotein, LDL: low-density lipoprotein, IDL: intermediate-density lipoprotein, HDL: high-density lipoprotein, c: cholesterol, DAG: diglycerides, TG: triglycerides, P: 1982 Pelotas Birth Cohort, B: British Women Heart and Health Study, W: Whitehall II Study, U: UKCTOCS nested case-control study, , A: The Avon Longitudinal Study of Children and Parents – mothers' cohort, M: Metabolomics consortium, SD units: standard deviation units, CI: confidence interval.

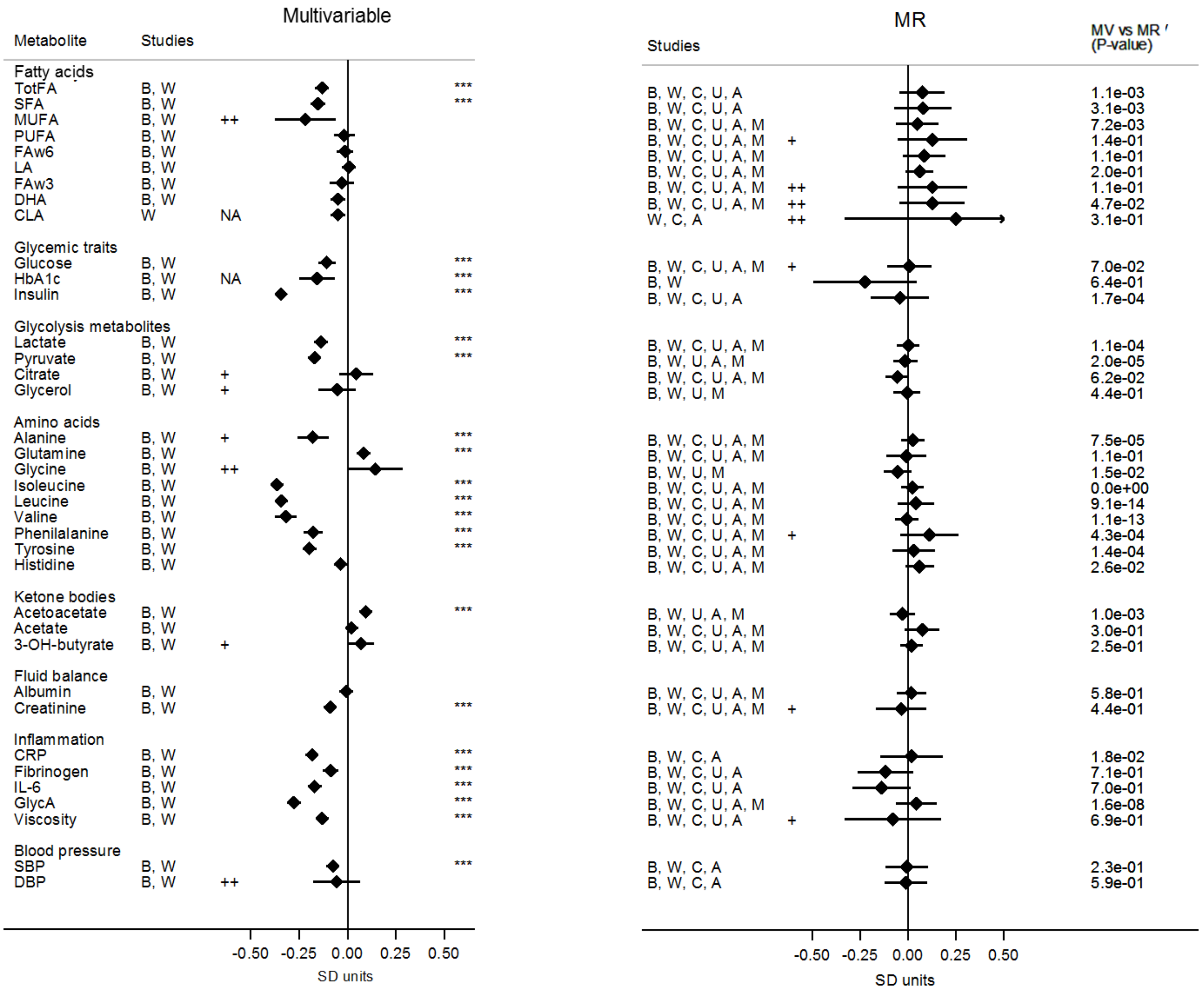

Supplementary figure 6. Association of multiple metabolic measures with blood adiponectin levels from observational and Mendelian randomization analysis restricted to individuals of European ancestry.

Values are expressed as units of standardized log metabolite concentration (and 95% CI) per 1 unit increment of standardized log adiponectin levels. P-values for the association between adiponectin and metabolites are indicated by three asterisks ("***") if lower than Bonferroni-adjusted threshold (P-value < 0.0019). Heterogeneity was considered substantial if I^2 = 50-75% ("+") or very high if I^2 > 75% ("++"). P-values for the comparison between multivariable and Mendelian randomization estimates are displayed in the column "MR vs MV (P-value)". Metabolic measures were adjusted for age, sex, and, if applicable, place of recruitment (BWHHS and UKCTOCS) or principal components of genomic ancestry (PEL82 and some studies contributing to Metabolomics consortium) and the resulting residuals were transformed to normal distribution by inverse rank-based normal transformation. TotFA: total fatty acids, SFA: saturated fatty acid, MUFA: monounsaturated fatty acid, PUFA: polyunsaturated fatty acids, FAw6: omega-6 fatty acid, LA: linoleic acid, FAw3: omega-3 fatty acid, DHA: docosaexaenoic acid, CLA: conjugated linoleic acids, HbA1c: glycated haemoglobin, CRP: c-reactive protein, IL-6: interleukin-6, GlycA: glycoprotein acetyls, SBP: systolic blood pressure, DBP: diastolic blood pressure, P: 1982 Pelotas Birth Cohort, B: British Women Heart and Health Study, W: Whitehall II Study, U: UKCTOCS nested case-control study, A: The Avon Longitudinal Study of Children and Parents – mothers' cohort, M: Metabolomics consortium, SD units: standard deviation units, CI: confidence interval.

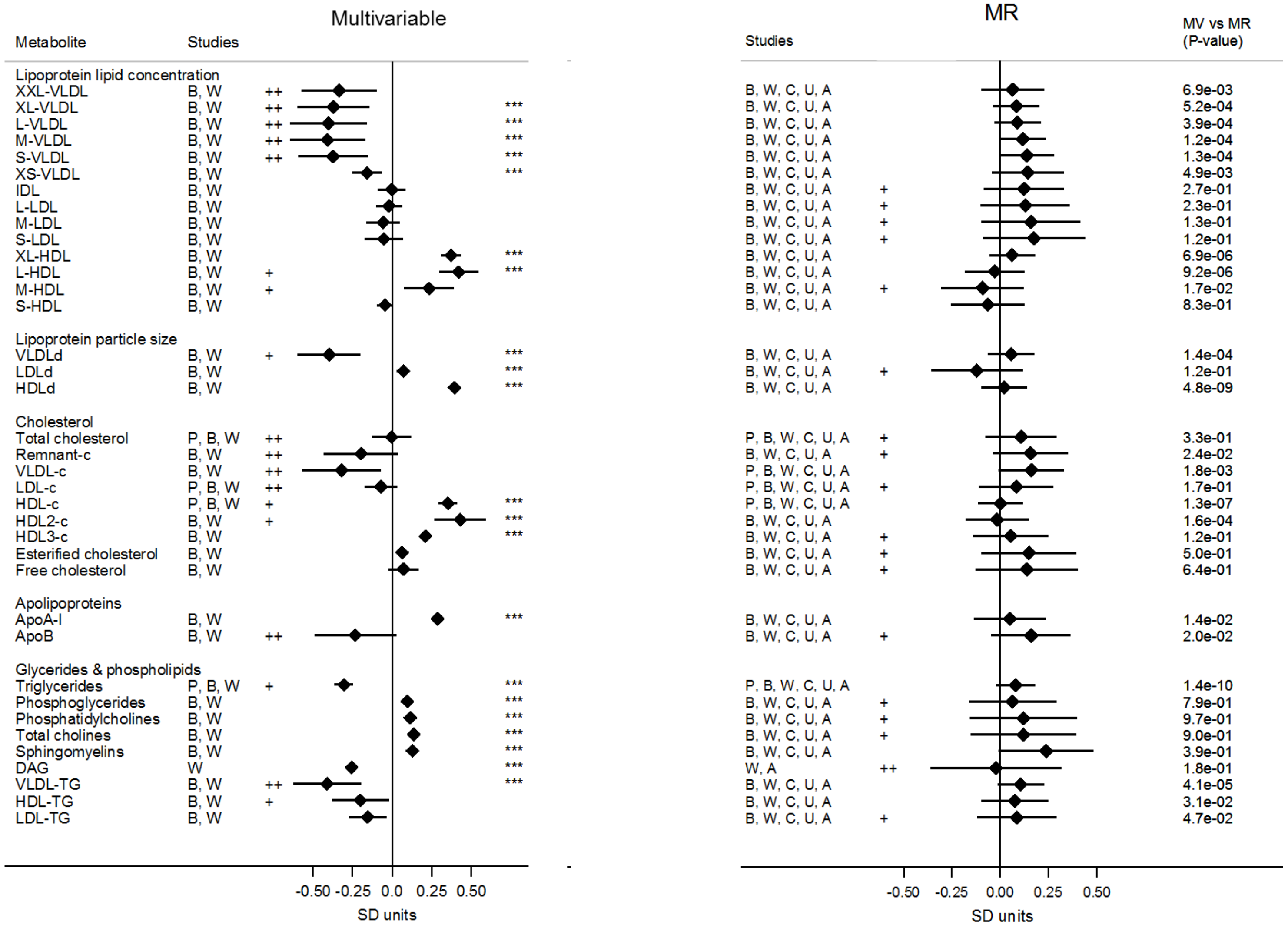

Supplementary figure 7. Association of lipoprotein traits with blood adiponectin levels from observational and Mendelian randomization (MR) analysis among younger individuals (< 65 years old) free from cardiovascular disease.

Values are expressed as units of standardized log metabolite concentration (and 95% CI) per 1 unit increment of standardized log adiponectin levels. P-values for the association between adiponectin and metabolites are indicated by three asterisks ("***") if lower than Bonferroni-adjusted threshold (P-value < 0.0019). Heterogeneity was considered substantial if I^2 = 50-75% ("+") or very high if I^2 > 75% ("++"). P-values for the comparison between multivariable and Mendelian randomization estimates are displayed in the column "MR vs MV (P-value)". Metabolic measures were adjusted for age, sex, and, if applicable, place of recruitment (BWHHS and UKCTOCS) or principal components of genomic ancestry (PEL82 and some studies contributing to Metabolomics consortium) and the resulting residuals were transformed to normal distribution by inverse rank-based normal transformation. XXL: extremely large, XL: very large, L: large, M: medium, S: small, XS: very small, VLDL: very low-density lipoprotein, LDL: low-density lipoprotein, IDL: intermediate-density lipoprotein, HDL: high-density lipoprotein, c: cholesterol, DAG: diglycerides, TG: triglycerides, P: 1982 Pelotas Birth Cohort, B: British Women Heart and Health Study, W: Whitehall II Study, U: UKCTOCS nested case-control study, A: The Avon Longitudinal Study of Children and Parents – mothers' cohort, SD units: standard deviation units, CI: confidence interval.

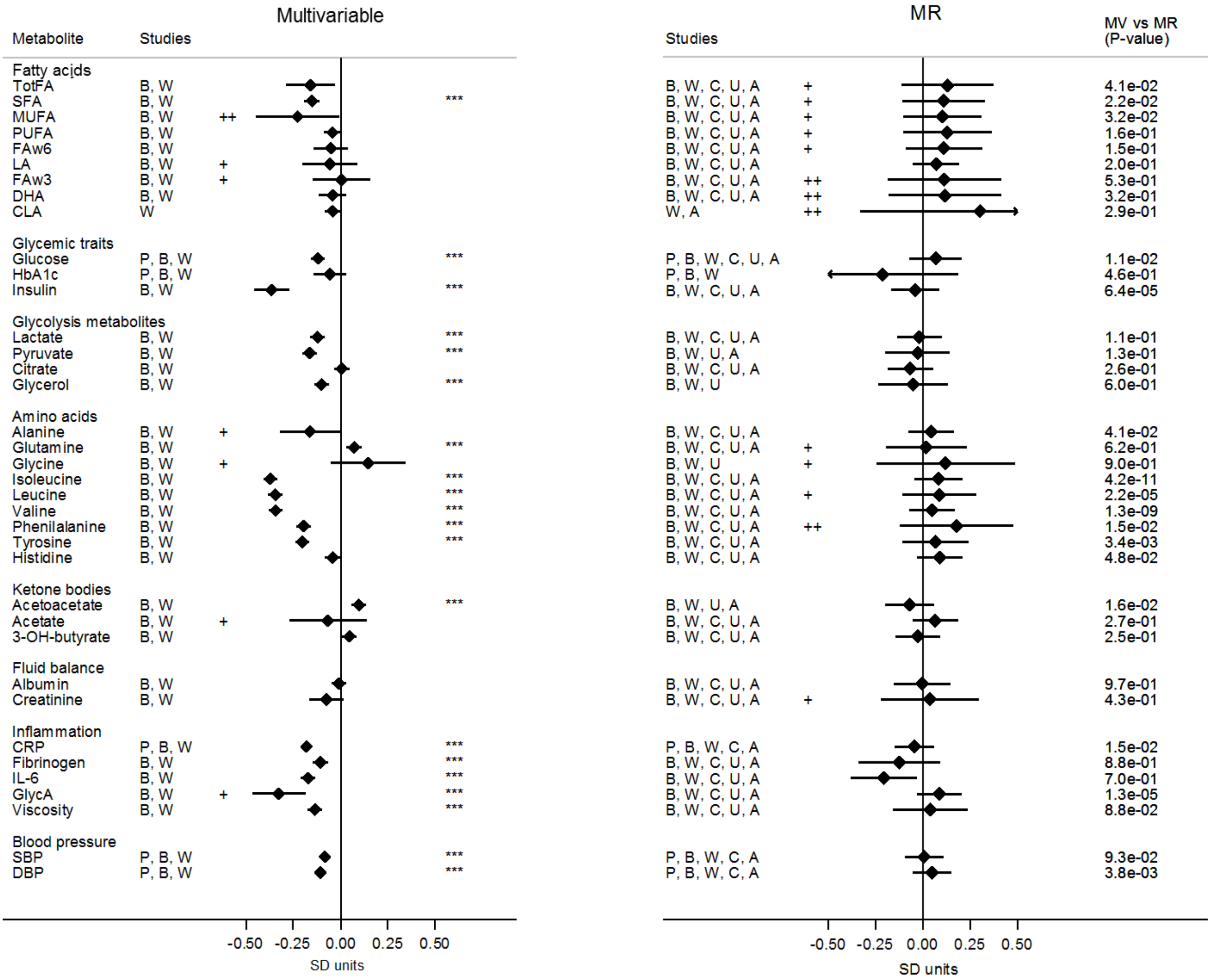

Supplementary figure 8. Association of multiple metabolic measures with blood adiponectin levels from observational and Mendelian randomization analysis among younger individuals (< 65 years old) free from cardiovascular disease.

Values are expressed as units of standardized log metabolite concentration (and 95% CI) per 1 unit increment of standardized log adiponectin levels. P-values for the association between adiponectin and metabolites are indicated by three asterisks ("***") if lower than Bonferroni-adjusted threshold (P-value < 0.0019). Heterogeneity was considered substantial if I^2 = 50-75% ("+") or very high if I^2 > 75% ("++"). P-values for the comparison between multivariable and Mendelian randomization estimates are displayed in the column "MR vs MV (P-value)". Metabolic measures were adjusted for age, sex, and, if applicable, place of recruitment (BWHHS and UKCTOCS) or principal components of genomic ancestry (PEL82 and some studies contributing to Metabolomics consortium) and the resulting residuals were transformed to normal distribution by inverse rank-based normal transformation. TotFA: total fatty acids, SFA: saturated fatty acid, MUFA: monounsaturated fatty acid, PUFA: polyunsaturated fatty acids, FAw6: omega-6 fatty acid, LA: linoleic acid, FAw3: omega-3 fatty acid, DHA: docosaexaenoic acid, CLA: conjugated linoleic acids, HbA1c: glycated haemoglobin, CRP: c-reactive protein, IL-6: interleukin-6, GlycA: glycoprotein acetyls, SBP: systolic blood pressure, DBP: diastolic blood pressure, P: 1982 Pelotas Birth Cohort, B: British Women Heart and Health Study, W: Whitehall II Study, U: UKCTOCS nested case-control study, A: The Avon Longitudinal Study of Children and Parents – mothers' cohort, M: Metabolomics consortium, SD units: standard deviation units, CI: confidence interval.

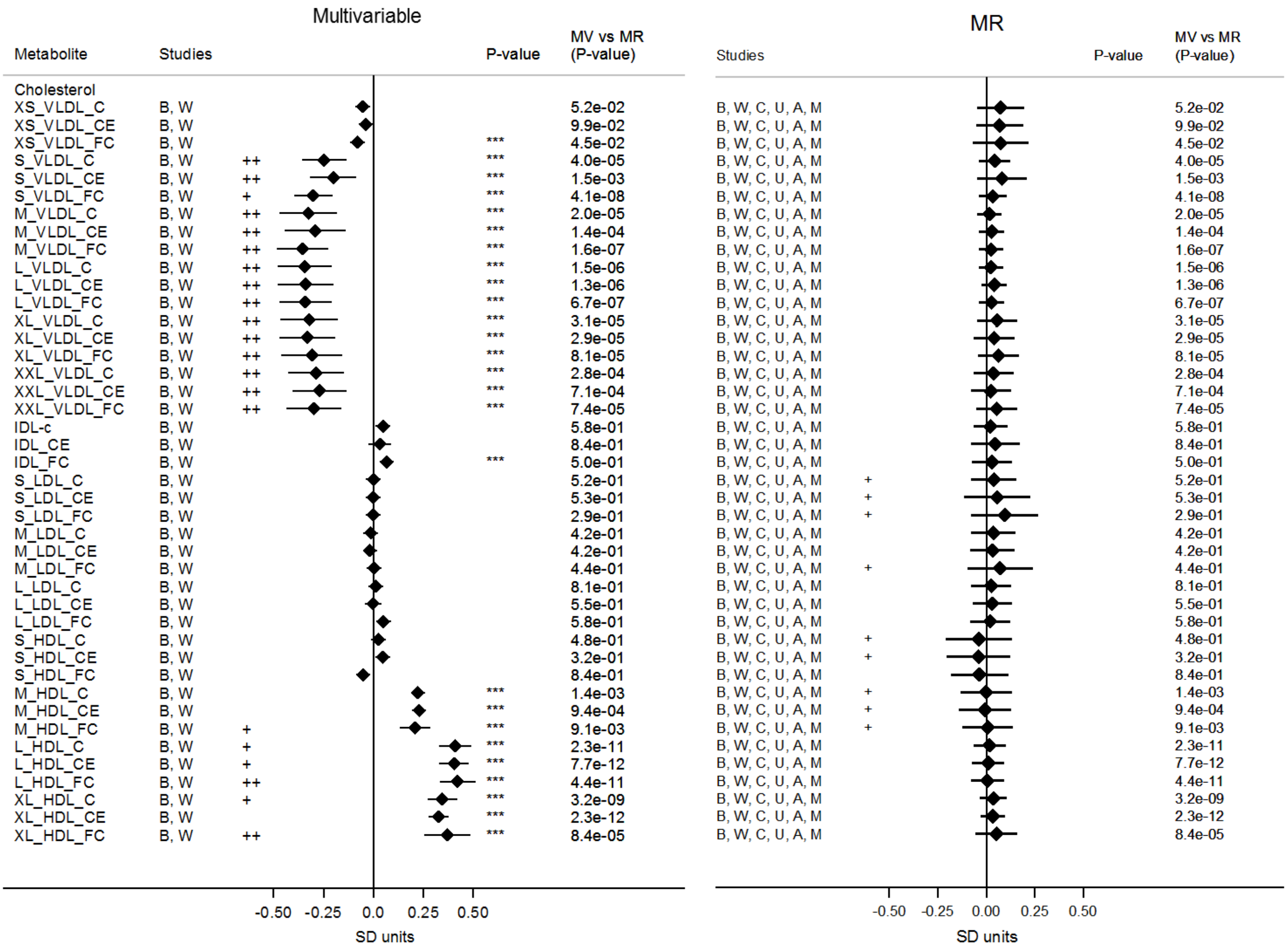

Supplementary figure 9. Association of metabolic measures not included in the main results with blood adiponectin levels from observational and Mendelian randomization analysis

Values are expressed as units of standardized log metabolite concentration (and 95% CI) per 1 unit increment of standardized log adiponectin levels. P-values for the association between adiponectin and metabolites are indicated by three asterisks ("***") if lower than Bonferroni-adjusted threshold (P-value < 0.0019). Heterogeneity was considered substantial if I^2 = 50-75% ("+") or very high if I^2 > 75% ("++"). P-values for the comparison between multivariable and Mendelian randomization estimates are displayed in the column "MR vs MV (P-value)". Metabolic measures were adjusted for age, sex, and, if applicable, place of recruitment (BWHHS and UKCTOCS) or principal components of genomic ancestry (PEL82 and some studies contributing to Metabolomics consortium) and the resulting residuals were transformed to normal distribution by inverse rank-based normal transformation. XXL: extremely large, XL: very large, L: large, M: medium, S: small, XS: very small, VLDL: very low-density lipoprotein, LDL: low-density lipoprotein, IDL: intermediate-density lipoprotein, HDL: high-density lipoprotein, c: cholesterol, DAG: diglycerides, TG: triglycerides, C: cholesterol, CE: cholesterol esters, FC: free cholesterol, P: 1982 Pelotas Birth Cohort, B: British Women Heart and Health Study, W: Whitehall II Study, U: UKCTOCS nested case-control study, A: The Avon Longitudinal Study of Children and Parents – mothers' cohort, SD units: standard deviation units, CI: confidence interval.

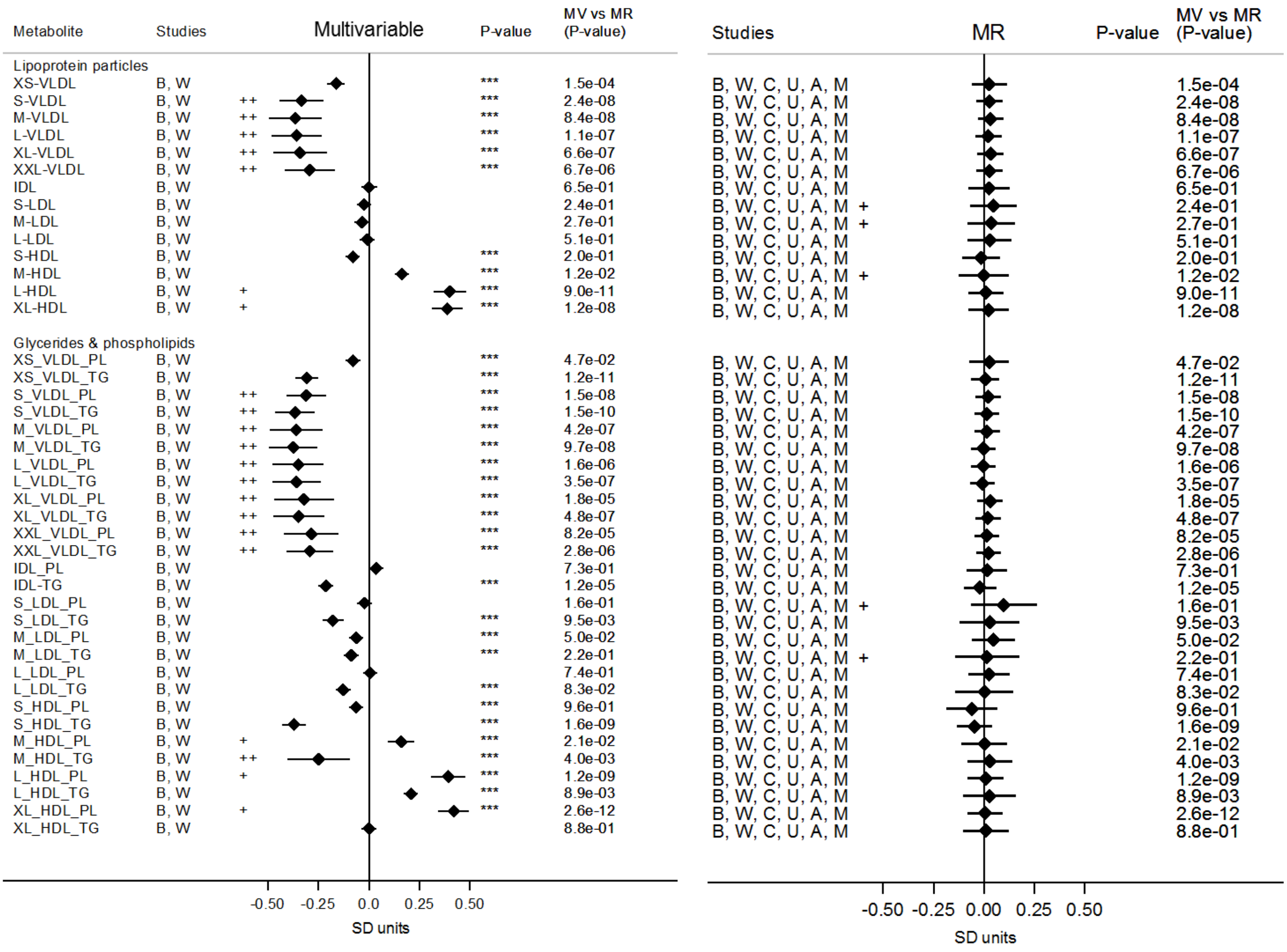

Supplementary figure 10. Association of metabolic measures not included in the main results with blood adiponectin levels from observational and Mendelian randomization analysis

Values are expressed as units of standardized log metabolite concentration (and 95% CI) per 1 unit increment of standardized log adiponectin levels. P-values for the association between adiponectin and metabolites are indicated by three asterisks ("***") if lower than Bonferroni-adjusted threshold (P-value < 0.0019). Heterogeneity was considered substantial if I^2 = 50-75% ("+") or very high if I^2 > 75% ("++"). P-values for the comparison between multivariable and Mendelian randomization estimates are displayed in the column "MR vs MV (P-value)". Metabolic measures were adjusted for age, sex, and, if applicable, place of recruitment (BWHHS and UKCTOCS) or principal components of genomic ancestry (PEL82 and some studies contributing to Metabolomics consortium) and the resulting residuals were transformed to normal distribution by inverse rank-based normal transformation. XXL: extremely large, XL: very large, L: large, M: medium, S: small, XS: very small, VLDL: very low-density lipoprotein, LDL: low-density lipoprotein, IDL: intermediate-density lipoprotein, HDL: high-density lipoprotein, c: cholesterol, DAG: diglycerides, TG: triglycerides, PL: phospholipids, P: 1982 Pelotas Birth Cohort, B: British Women Heart and Health Study, W: Whitehall II Study, U: UKCTOCS nested case-control study, A: The Avon Longitudinal Study of Children and Parents – mothers' cohort, SD units: standard deviation units, CI: confidence interval.

Supplementary table 1A – Estimated power in Multivariable regression analysis

Exposure	Outcome	Sample size*	Type-I error rate	Effect estimate†	Power
Adiponectin	Metabolic measure	3,006	0.05	0.20	100%
Adiponectin	Metabolic measure	3,006	0.05	0.10	100%
Adiponectin	Metabolic measure	3,006	0.05	0.05	78%

* Median sample size

† Considering the true underlying causal association is unknown, a range of values (in standard deviation units) was used.

Supplementary table 1B – Estimated power in Mendelian randomization analysis

Exposure	Outcome	Sample size*	Type-I error rate	Effect estimate†	Instrument strength (R^2) ‡	Power§
Adiponectin	Metabolic measure	23,884	0.05	0.20	0.04	88%
Adiponectin	Metabolic measure	23,884	0.05	0.10	0.04	87%
Adiponectin	Metabolic measure	23,884	0.05	0.05	0.04	34%

* Median sample size used for estimating SNP-outcome association

† Considering the true underlying causal association is unknown, a range of values (in standard deviation units) was used.

‡ Instrument strength relates to the proportion of variance in the exposure explained by the instrument (R^2). This was calculated by the sum of R^2 from each 4 SNPs in the instrument. The formula used to estimate R^2 for each SNP is detailed in Supplementary methods.

§ We have estimated power for our Mendelian randomization analyses using the online calculator tool (http://cnsgenomics.com/shiny/mRnd/) and assuming a range of effect sizes for the potential underlying causal association between exposure and outcome.

Supplementary table 2 – Total sample size per metabolite of multivariable and Mendelian randomization analysis

Metabolic measure	Group	N (multivariable analysis)	N (Mendelian randomization analysis)
Acetoacetate	Ketone bodies	3008	28225
Acetate	Ketone bodies	3008	34923
Alanine	Amino acids	3006	34969
Albumin	Fluid balance	3007	29139
ApoA-I	Apolipoproteins	3008	28326
ApoB	Apolipoproteins	3008	30868
3-OH-butyrate	Ketone bodies	3003	34295
Citrate	Glycolysis metabolites	3003	34937
CLA	Fatty acids	2498	5549
Creatinine	Fluid balance	2939	34756
CRP	Inflammation	5826	11039
DAG	Glycerides & phospholipids	2470	6521
DBP	Blood pressure	5909	11821
DHA	Fatty acids	2958	23497
Esterified cholesterol	Cholesterol	2960	23498
FAw3	Fatty acids	2959	22985
FAw6	Fatty acids	2958	23504
Fibrinogen	Inflammation	3029	5675
Free cholesterol	Cholesterol	2959	23497
Glucose	Glycemic traits	5720	37545
Glutamine	Amino acids	3006	34570
Glycerol	Glycolysis metabolites	2975	26391
Glycine	Amino acids	2954	24919
GlycA	Inflammation	3009	29446
HbA1c	Glycemic traits	3239	4647
HDL2-c	Cholesterol	3008	10183
HDL3-c	Cholesterol	3008	10183
HDL-c	Cholesterol	5762	30202
HDLd	Lipoprotein particle size	3008	29452
HDL-TG	Glycerides & phospholipids	3008	10183
Histidine	Amino acids	2976	29387
IDL	Lipoprotein lipid concentration	3008	29452
IL-6	Inflammation	3106	5585
Isoleucine	Amino acids	3008	34947
Insulin	Glycemic traits	3155	8148
LA	Fatty acids	2958	23524
Lactate	Glycolysis metabolites	3009	35047
LDL-c	Cholesterol	5762	34492
LDLd	Lipoprotein particle size	3008	29452
LDL-TG	Glycerides & phospholipids	3008	10183
Leucine	Amino acids	3009	34905
L-HDL	Lipoprotein lipid concentration	3009	29458
L-LDL	Lipoprotein lipid concentration	3009	29458
L-VLDL	Lipoprotein lipid concentration	3009	29146
M-HDL	Lipoprotein lipid concentration	3009	29458
M-LDL	Lipoprotein lipid concentration	3009	29458
MUFA	Fatty acids	2958	23522
M-VLDL	Lipoprotein lipid concentration	3009	29458
Phosphatidylcholines	Glycerides & phospholipids	2960	23502
Phenilalanine	Amino acids	3004	32833
PUFA	Fatty acids	2958	10003
Pyruvate	Glycolysis metabolites	3003	31327
Remnant-c	Cholesterol	3008	10183
SBP	Blood pressure	5909	11822
Total cholesterol	Cholesterol	5762	30763
Triglycerides	Glycerides & phospholipids	5762	34478
SFA	Fatty acids	2957	9988
S-HDL	Lipoprotein lipid concentration	3009	29458
S-LDL	Lipoprotein lipid concentration	3009	29458
Sphingomyelins	Glycerides & phospholipids	2959	20854
S-VLDL	Lipoprotein lipid concentration	3009	29458
Total cholines	Glycerides & phospholipids	2960	10007
TotFA	Fatty acids	2959	23503
Phosphoglycerides	Glycerides & phospholipids	2960	23521
Tyrosine	Amino acids	2994	35078
Valine	Amino acids	3009	35069
Viscosity	Inflammation	3094	4807
VLDL-c	Cholesterol	3008	10183
VLDLd	Lipoprotein particle size	3008	29452
VLDL-TG	Glycerides & phospholipids	3008	10183
XL-HDL	Lipoprotein lipid concentration	3009	29458
XL-VLDL	Lipoprotein lipid concentration	3009	29458
XS-VLDL	Lipoprotein lipid concentration	3009	29458
XXL-VLDL	Lipoprotein lipid concentration	3009	29146

Supplementary table 3 – Mean (and 95% confidence interval) of metabolic measures for each study that contributed with individual level data

		PEL82		BWHHS		WHII		CaPS		UKCTOCS		ALSPAC-M	
Metabolite	Units	N	Mean (95% CI)	N	Mean (95% CI)	N	Mean (95% CI)	N	Mean (95% CI)	N	Mean (95% CI)	N	Mean (95% CI)
Acetoacetate	mmol/L			3774	0.07 (0.07; 0.07)	4639	0.06 (0.06; 0.06)			4812	0.03 (0.03; 0.03)	4134	0.03 (0.03; 0.03)
Acetate	mmol/L			3774	0.04 (0.04; 0.04)	4638	0.07 (0.07; 0.07)	1223	0.09 (0.09; 0.09)	4811	0.05 (0.05; 0.05)	4135	0.06 (0.06; 0.07)
Alanine	mmol/L			3774	0.33 (0.33; 0.34)	4637	0.42 (0.42; 0.42)	1223	0.39 (0.38; 0.39)	4811	0.52 (0.52; 0.52)	4135	0.25 (0.25; 0.25)
Albumin	signal area			3779	0.1 (0.1; 0.1)	4638	0.1 (0.1; 0.1)	1223	0.08 (0.08; 0.09)	4811	0.09 (0.09; 0.09)	4138	0.09 (0.09; 0.09)
ApoA-I	g/L			3777	1.73 (1.72; 1.74)	4639	1.59 (1.59; 1.6)	1223	1.25 (1.24; 1.25)	4810	1.69 (1.68; 1.69)	4138	1.69 (1.68; 1.69)
ApoB	g/L			3777	1.15 (1.14; 1.16)	4639	0.98 (0.97; 0.98)	1223	0.94 (0.93; 0.96)	4810	0.95 (0.94; 0.96)	4138	0.86 (0.86; 0.87)
3-OH-butyrate	mmol/L			3773	0.19 (0.19; 0.2)	4631	0.15 (0.15; 0.16)	1205	0.12 (0.12; 0.13)	4797	0.18 (0.18; 0.18)	4129	0.11 (0.11; 0.12)
Citrate	mmol/L			3774	0.12 (0.12; 0.12)	4631	0.13 (0.13; 0.13)	1220	0.11 (0.11; 0.12)	4812	0.13 (0.13; 0.13)	4134	0.09 (0.09; 0.09)
CLA	mmol/L					4557	0.05 (0.05; 0.05)	104	0.03 (0.02; 0.03)			3950	0.02 (0.02; 0.02)
Creatinine	mmol/L			3469	0.06 (0.06; 0.06)	4592	0.08 (0.08; 0.08)	1222	0.07 (0.07; 0.07)	4803	0.06 (0.06; 0.06)	4134	0.06 (0.06; 0.06)
CRP	mg/dL	3524	3.93 (3.75; 4.11)	3709	3.49 (3.3; 3.68)	4712	2.07 (1.96; 2.18)	841	2.73 (2.48; 2.98)			4160	2.3 (2.13; 2.48)
DAG	mmol/L					4512	0.02 (0.02; 0.02)	1184	0.02 (0.02; 0.02)			3830	0.01 (0.01; 0.01)
DBP	mmHg	3580	75.45 (75.15; 75.76)	3964	79.42 (79.05; 79.79)	4874	77.45 (77.16; 77.74)	1206	84.78 (84.12; 85.43)			4570	72.72 (72.42; 73.02)
DHA	mmol/L			3769	0.3 (0.3; 0.3)	4558	0.19 (0.19; 0.2)	1219	0.12 (0.11; 0.12)	4797	0.19 (0.19; 0.19)	3954	0.15 (0.14; 0.15)
Esterified cholesterol	mmol/L			3769	4.22 (4.19; 4.25)	4561	3.66 (3.64; 3.68)	1220	2.72 (2.68; 2.76)	4797	3.56 (3.54; 3.58)	3953	3.27 (3.26; 3.29)
FAw3	mmol/L			3769	0.73 (0.72; 0.74)	4559	0.53 (0.53; 0.54)	1219	0.33 (0.33; 0.34)	4797	0.54 (0.53; 0.54)	3954	0.44 (0.43; 0.44)
FAw6	mmol/L			3769	4.63 (4.6; 4.66)	4558	4.14 (4.12; 4.16)	1219	3.03 (2.99; 3.07)	4797	3.97 (3.94; 3.99)	3953	3.71 (3.69; 3.73)
Fibrinogen	mmol/L			3817	3.45 (3.43; 3.47)	4620	3.02 (3; 3.04)	847	3.04 (2.99; 3.1)				
Free cholesterol	mmol/L			3769	1.77 (1.75; 1.78)	4560	1.42 (1.41; 1.43)	1220	1.2 (1.19; 1.22)	4797	1.39 (1.38; 1.4)	3953	1.36 (1.35; 1.37)
Glucose	mmol/L	3524	4.97 (4.92; 5.02)	3774	4.94 (4.89; 4.98)	4568	5.15 (5.12; 5.18)	1222	4.07 (4; 4.14)	4778	2.64 (2.58; 2.69)	4133	4.48 (4.45; 4.51)
Glutamine	mmol/L			3773	0.49 (0.49; 0.5)	4636	0.61 (0.61; 0.61)	1166	0.39 (0.39; 0.39)	4798	0.52 (0.52; 0.52)	4134	0.47 (0.47; 0.47)
Glycerol	mmol/L			3699	0.13 (0.13; 0.14)	4600	0.1 (0.1; 0.1)			4777	0.11 (0.11; 0.11)		
Glycine	mmol/L			3774	0.3 (0.3; 0.31)	4559	0.29 (0.29; 0.29)			4808	0.37 (0.37; 0.38)		
GlycA	mmol/L			3774	1.6 (1.59; 1.62)	4640	1.44 (1.43; 1.44)	1223	1.33 (1.32; 1.35)	4812	1.59 (1.58; 1.6)	4135	1.25 (1.24; 1.25)
HbA1c	%	3537	5.12 (5.1; 5.13)	3703	5.01 (4.98; 5.03)								
HDL2-c	mmol/L			3777	1.15 (1.13; 1.16)	4639	1.04 (1.03; 1.05)	1223	0.47 (0.45; 0.48)	4810	1.09 (1.08; 1.1)	4138	1.15 (1.14; 1.16)
HDL3-c	mmol/L			3777	0.52 (0.52; 0.52)	4639	0.48 (0.48; 0.48)	1223	0.42 (0.42; 0.43)	4810	0.55 (0.55; 0.55)	4138	0.55 (0.55; 0.55)
HDL-c	mmol/L	3524	1.52 (1.5; 1.53)	3777	1.67 (1.65; 1.68)	4639	1.52 (1.51; 1.53)	1223	0.89 (0.88; 0.9)	4810	1.64 (1.63; 1.66)	4138	1.7 (1.69; 1.71)
HDLd	nm			3777	9.98 (9.97; 9.99)	4639	10.03 (10.03; 10.04)	1223	9.57 (9.56; 9.58)	4810	10.01 (10; 10.02)	4138	10.09 (10.09; 10.1)
HDL-TG	mmol/L			3777	0.17 (0.17; 0.18)	4639	0.14 (0.14; 0.14)	1223	0.15 (0.14; 0.15)	4810	0.19 (0.19; 0.19)	4138	0.14 (0.14; 0.14)
Histidine	mmol/L			3774	0.06 (0.06; 0.06)	4585	0.08 (0.07; 0.08)	1223	0.06 (0.06; 0.06)	4807	0.08 (0.08; 0.08)	4134	0.06 (0.06; 0.06)
IDL	mmol/L			3777	1.53 (1.51; 1.54)	4639	1.33 (1.32; 1.34)	1223	1.03 (1.01; 1.04)	4810	1.17 (1.16; 1.18)	4138	1.12 (1.11; 1.13)
IL-6	pg/mL			3815	3.23 (3.1; 3.37)	4732	1.82 (1.77; 1.86)	701	3.38 (2.16; 4.61)				
Isoleucine	mmol/L			3774	0.06 (0.06; 0.06)	4639	0.06 (0.06; 0.06)	1222	0.06 (0.06; 0.07)	4811	0.07 (0.07; 0.07)	4133	0.03 (0.03; 0.03)
Insulin	mIU/L			3831	9.58 (8.78; 10.38)	4809	9.65 (9.29; 10)	608	5.85 (5.46; 6.23)			4092	5.24 (5.08; 5.4)
LA	mmol/L			3769	3.76 (3.74; 3.79)	4558	3.37 (3.36; 3.39)	1219	2.5 (2.46; 2.54)	4797	3.15 (3.13; 3.17)	3953	2.93 (2.91; 2.94)
Lactate	mmol/L			3774	1.64 (1.63; 1.66)	4640	1.91 (1.9; 1.93)	1223	1.74 (1.72; 1.76)	4812	5.58 (5.53; 5.63)	4135	0.8 (0.79; 0.81)
LDL-c	mmol/L	3524	2.83 (2.8; 2.85)	3777	2.42 (2.4; 2.44)	4639	1.88 (1.86; 1.89)	1223	1.6 (1.57; 1.63)	4810	1.8 (1.79; 1.82)	4138	1.56 (1.54; 1.57)
LDLd	mmol/L			3777	23.52 (23.52; 23.53)	4639	23.68 (23.68; 23.69)	1223	23.51 (23.5; 23.51)	4810	23.45 (23.45; 23.45)	4138	23.62 (23.62; 23.62)
LDL-TG	mmol/L			3777	0.28 (0.27; 0.28)	4639	0.19 (0.19; 0.19)	1223	0.17 (0.17; 0.17)	4810	0.26 (0.26; 0.26)	4138	0.17 (0.17; 0.17)
Leucine	mmol/L			3774	0.07 (0.07; 0.07)	4639	0.09 (0.09; 0.09)	1223	0.08 (0.08; 0.08)	4812	0.1 (0.1; 0.1)	4134	0.05 (0.05; 0.05)
L-HDL	mmol/L			3780	0.82 (0.81; 0.84)	4641	0.81 (0.8; 0.82)	1225	0.26 (0.25; 0.27)	4813	0.94 (0.93; 0.95)	4138	1 (0.99; 1.01)
L-LDL	mmol/L			3780	1.83 (1.82; 1.85)	4641	1.47 (1.46; 1.48)	1225	1.22 (1.2; 1.24)	4813	1.39 (1.38; 1.4)	4138	1.24 (1.22; 1.25)
L-VLDL	mmol/L			3780	0.33 (0.32; 0.34)	4641	0.19 (0.19; 0.2)	1225	0.44 (0.42; 0.46)	4813	0.33 (0.32; 0.34)	4138	0.17 (0.16; 0.17)
M-HDL	mmol/L			3780	1.04 (1.03; 1.05)	4641	0.99 (0.98; 0.99)	1225	0.62 (0.61; 0.63)	4813	0.95 (0.94; 0.96)	4138	1.03 (1.02; 1.03)
M-LDL	mmol/L			3780	1.08 (1.07; 1.09)	4641	0.82 (0.81; 0.82)	1225	0.73 (0.72; 0.74)	4813	0.84 (0.83; 0.84)	4138	0.69 (0.68; 0.69)
MUFA	mmol/L			3769	3.13 (3.1; 3.17)	4558	3 (2.98; 3.02)	1219	2.72 (2.66; 2.77)	4761	3.6 (3.57; 3.63)	3951	2.81 (2.79; 2.84)
M-VLDL	mmol/L			3780	0.71 (0.69; 0.72)	4641	0.53 (0.52; 0.54)	1225	0.83 (0.81; 0.86)	4813	0.68 (0.67; 0.69)	4138	0.41 (0.4; 0.42)
Phosphatidylcholines	mmol/L			3769	2.49 (2.47; 2.5)	4561	2.01 (2; 2.02)	1219	1.56 (1.55; 1.58)	4713	2.1 (2.09; 2.11)	3930	2.03 (2.02; 2.04)
Phenilalanine	mmol/L			3774	0.08 (0.08; 0.08)	4633	0.08 (0.08; 0.08)	1223	0.07 (0.07; 0.08)	4812	0.1 (0.1; 0.1)	4132	0.04 (0.04; 0.04)
PUFA	mmol/L			3769	5.36 (5.32; 5.4)	4558	4.67 (4.64; 4.69)	1219	3.36 (3.32; 3.41)	4797	4.5 (4.48; 4.53)	3953	4.15 (4.13; 4.17)
Pyruvate	mmol/L			3772	0.1 (0.1; 0.1)	4633	0.09 (0.09; 0.09)			4808	0.23 (0.22; 0.24)	4135	0.09 (0.09; 0.09)
Remnant-c	mmol/L			3777	1.9 (1.88; 1.91)	4639	1.69 (1.68; 1.7)	1223	1.44 (1.41; 1.46)	4810	1.5 (1.48; 1.51)	4138	1.38 (1.37; 1.39)
SBP	mmHg	3580	121.29 (120.84; 121.74)	3964	147.11 (146.33; 147.9)	4874	122.85 (122.4; 123.31)	1207	146.19 (144.91; 147.47)			4570	118.46 (118.09; 118.82)
Total cholesterol	mmol/L	3524	4.94 (4.91; 4.98)	3777	5.98 (5.94; 6.03)	4639	5.08 (5.06; 5.11)	1223	3.93 (3.88; 3.98)	4810	4.94 (4.91; 4.97)	4138	4.63 (4.61; 4.66)
Triglycerides	mmol/L	3524	1.37 (1.33; 1.41)	3777	1.68 (1.65; 1.71)	4639	1.22 (1.21; 1.23)	1223	1.68 (1.64; 1.73)	4810	1.68 (1.66; 1.71)	4138	1.06 (1.04; 1.07)
SFA	mmol/L			3769	4.91 (4.88; 4.95)	4557	4.56 (4.54; 4.59)	1219	4.05 (3.99; 4.11)	4761	4.82 (4.79; 4.85)	3949	4.15 (4.13; 4.18)
S-HDL	mmol/L			3780	1.24 (1.23; 1.24)	4641	1.09 (1.08; 1.09)	1225	0.98 (0.97; 0.99)	4813	1.21 (1.2; 1.21)	4138	1.12 (1.12; 1.13)
S-LDL	mmol/L			3780	0.68 (0.68; 0.69)	4641	0.52 (0.51; 0.52)	1225	0.46 (0.45; 0.47)	4813	0.55 (0.54; 0.55)	4138	0.45 (0.44; 0.45)
Sphingomyelins	mmol/L			3769	0.61 (0.6; 0.61)	4560	0.5 (0.5; 0.5)	1220	0.45 (0.44; 0.45)	4786	0.5 (0.5; 0.5)		
S-VLDL	mmol/L			3780	0.79 (0.77; 0.8)	4641	0.66 (0.66; 0.67)	1225	0.71 (0.7; 0.72)	4813	0.74 (0.73; 0.75)	4138	0.55 (0.54; 0.55)
Total cholines	mmol/L			3769	2.95 (2.93; 2.97)	4561	2.41 (2.4; 2.42)	1220	1.84 (1.82; 1.87)	4797	2.46 (2.45; 2.47)	3953	2.35 (2.34; 2.36)
TotFA	mmol/L			3769	13.41 (13.32; 13.51)	4559	12.23 (12.17; 12.29)	1219	10.13 (9.98; 10.28)	4797	12.94 (12.86; 13.02)	3952	11.12 (11.05; 11.18)
Phosphoglycerides	mmol/L			3769	2.44 (2.42; 2.46)	4561	2.04 (2.03; 2.05)	1220	1.52 (1.5; 1.54)	4797	2 (1.99; 2.01)	3951	1.94 (1.92; 1.95)
Tyrosine	mmol/L			3774	0.05 (0.05; 0.05)	4615	0.06 (0.06; 0.06)	1222	0.05 (0.05; 0.05)	4805	0.07 (0.07; 0.07)	4132	0.05 (0.05; 0.05)
Valine	mmol/L			3774	0.17 (0.16; 0.17)	4640	0.2 (0.2; 0.2)	1221	0.16 (0.16; 0.17)	4810	0.2 (0.2; 0.2)	4133	0.14 (0.14; 0.15)
Viscosity	mPa.s.			3636	1.3 (1.3; 1.3)	4741	1.26 (1.25; 1.26)						
VLDL-c	mmol/L			3777	0.94 (0.93; 0.95)	4639	0.84 (0.83; 0.84)	1223	0.8 (0.79; 0.82)	4810	0.8 (0.79; 0.81)	4138	0.67 (0.66; 0.68)
VLDLd	mmol/L			3777	36.31 (36.27; 36.36)	4639	35.82 (35.79; 35.84)	1223	37.77 (37.7; 37.83)	4810	36.51 (36.47; 36.55)	4138	35.78 (35.74; 35.82)
VLDL-TG	mmol/L			3777	1.07 (1.04; 1.09)	4639	0.77 (0.75; 0.78)	1223	1.25 (1.21; 1.29)	4810	1.07 (1.05; 1.09)	4138	0.64 (0.63; 0.65)
XL-HDL	mmol/L			3780	0.52 (0.51; 0.53)	4641	0.47 (0.47; 0.48)	1225	0.16 (0.15; 0.17)	4813	0.51 (0.5; 0.52)	4138	0.55 (0.54; 0.56)
XL-VLDL	mmol/L			3780	0.08 (0.08; 0.08)	4641	0.04 (0.04; 0.04)	1225	0.11 (0.1; 0.11)	4813	0.08 (0.07; 0.08)	4138	0.04 (0.04; 0.04)
XS-VLDL	mmol/L			3780	0.69 (0.68; 0.69)	4641	0.64 (0.64; 0.65)	1225	0.49 (0.49; 0.5)	4813	0.59 (0.58; 0.59)	4138	0.54 (0.53; 0.54)
XXL-VLDL	mmol/L			3780	0.03 (0.03; 0.03)	4641	0.02 (0.02; 0.02)	1225	0.04 (0.04; 0.04)	4813	0.03 (0.03; 0.03)	4138	0.02 (0.02; 0.02)

Supplementary table 4A – Multivariable regression estimates for each contributing study

	PEL82	BWHHS	WHII
Metabolite	Mean SD units (95% CI)	Mean SD units (95% CI)	Mean SD units (95% CI)
3-OH-butyrate		0.12 (0.03; 0.2)	0.04 (0.01; 0.08)
Acetate		0.05 (-0.04; 0.14)	0.02 (-0.02; 0.05)
Acetoacetate		0.11 (0.02; 0.2)	0.09 (0.05; 0.13)
Alanine		-0.13 (-0.22; -0.04)	-0.21 (-0.25; -0.17)
Albumin		0.04 (-0.05; 0.12)	-0.01 (-0.05; 0.02)
ApoA-I		0.31 (0.22; 0.39)	0.27 (0.24; 0.31)
ApoB		-0.25 (-0.34; -0.16)	-0.11 (-0.15; -0.07)
CLA			-0.05 (-0.09; -0.01)
CRP	-0.17 (-0.2; -0.13)	-0.17 (-0.26; -0.08)	-0.19 (-0.22; -0.15)
Citrate		0.1 (0.01; 0.19)	0.01 (-0.03; 0.05)
Creatinine		-0.05 (-0.15; 0.05)	-0.1 (-0.13; -0.06)
DAG			-0.25 (-0.29; -0.21)
DBP	-0.1 (-0.14; -0.07)	0.01 (-0.08; 0.1)	-0.11 (-0.15; -0.07)
DHA		-0.01 (-0.1; 0.08)	-0.06 (-0.1; -0.02)
Esterified cholesterol		0.03 (-0.06; 0.13)	0.07 (0.03; 0.11)
FAw3		0.02 (-0.07; 0.11)	-0.05 (-0.09; -0.01)
FAw6		0.03 (-0.06; 0.13)	-0.02 (-0.06; 0.02)
Fibrinogen		-0.05 (-0.13; 0.04)	-0.1 (-0.14; -0.06)
Free cholesterol		0.03 (-0.06; 0.13)	0.1 (0.06; 0.14)
Glucose	-0.13 (-0.17; -0.09)	-0.06 (-0.15; 0.02)	-0.12 (-0.16; -0.08)
Glutamine		0.08 (-0.01; 0.17)	0.08 (0.04; 0.12)
GlycA		-0.3 (-0.39; -0.22)	-0.27 (-0.31; -0.24)
Glycerol		0.01 (-0.09; 0.1)	-0.1 (-0.13; -0.06)
Glycine		0.22 (0.13; 0.32)	0.08 (0.04; 0.12)
HDL-TG		-0.26 (-0.35; -0.17)	-0.14 (-0.17; -0.1)
HDL-c	0.31 (0.28; 0.35)	0.44 (0.35; 0.52)	0.34 (0.31; 0.38)
HDL2-c		0.44 (0.36; 0.53)	0.36 (0.32; 0.39)
HDL3-c		0.19 (0.1; 0.28)	0.19 (0.16; 0.23)
HDLd		0.48 (0.4; 0.56)	0.39 (0.35; 0.42)
HbA1c	-0.07 (-0.11; -0.04)	-0.16 (-0.25; -0.06)	
Histidine		-0.03 (-0.12; 0.06)	-0.04 (-0.08; 0)
IDL		-0.03 (-0.12; 0.07)	0.02 (-0.02; 0.06)
IL-6		-0.15 (-0.24; -0.06)	-0.17 (-0.21; -0.14)
Insulin		-0.38 (-0.46; -0.3)	-0.34 (-0.37; -0.3)
Isoleucine		-0.38 (-0.46; -0.29)	-0.36 (-0.4; -0.33)
L-HDL		0.45 (0.37; 0.53)	0.37 (0.33; 0.4)
L-LDL		-0.04 (-0.13; 0.06)	0.01 (-0.03; 0.04)
L-VLDL		-0.43 (-0.51; -0.34)	-0.3 (-0.33; -0.26)
LA		0.04 (-0.05; 0.14)	0 (-0.04; 0.04)
LDL-TG		-0.15 (-0.24; -0.05)	-0.13 (-0.16; -0.09)
LDL-c	-0.12 (-0.16; -0.09)	-0.03 (-0.12; 0.06)	0.01 (-0.03; 0.05)
LDLd		0.05 (-0.05; 0.14)	0.08 (0.04; 0.12)
Lactate		-0.16 (-0.25; -0.08)	-0.13 (-0.17; -0.09)
Leucine		-0.34 (-0.42; -0.26)	-0.34 (-0.38; -0.31)
M-HDL		0.21 (0.12; 0.29)	0.16 (0.13; 0.2)
M-LDL		-0.06 (-0.15; 0.03)	-0.02 (-0.06; 0.02)
M-VLDL		-0.44 (-0.52; -0.35)	-0.3 (-0.34; -0.26)
MUFA		-0.31 (-0.39; -0.22)	-0.14 (-0.18; -0.1)
PUFA		0.03 (-0.06; 0.12)	-0.03 (-0.07; 0.01)
Phenilalanine		-0.13 (-0.22; -0.05)	-0.19 (-0.23; -0.16)
Phosphatidylcholines		0.11 (0.03; 0.2)	0.11 (0.07; 0.15)
Phosphoglycerides		0.08 (-0.01; 0.17)	0.09 (0.05; 0.13)
Pyruvate		-0.17 (-0.26; -0.09)	-0.17 (-0.21; -0.13)
Remnant-c		-0.23 (-0.32; -0.14)	-0.09 (-0.13; -0.05)
S-HDL		-0.08 (-0.17; 0.01)	-0.06 (-0.1; -0.03)
S-LDL		-0.05 (-0.14; 0.04)	-0.01 (-0.05; 0.03)
S-VLDL		-0.39 (-0.48; -0.3)	-0.28 (-0.31; -0.24)
SBP	-0.09 (-0.13; -0.05)	-0.08 (-0.17; 0.01)	-0.08 (-0.11; -0.04)
SFA		-0.15 (-0.24; -0.06)	-0.15 (-0.19; -0.11)
Sphingomyelins		0.19 (0.1; 0.28)	0.13 (0.09; 0.17)
TotFA		-0.17 (-0.27; -0.08)	-0.12 (-0.16; -0.09)
Total cholesterol	-0.07 (-0.11; -0.03)	0.04 (-0.06; 0.13)	0.08 (0.04; 0.12)
Total cholines		0.14 (0.05; 0.23)	0.13 (0.09; 0.17)
Triglycerides	-0.27 (-0.31; -0.24)	-0.41 (-0.49; -0.32)	-0.3 (-0.34; -0.26)
Tyrosine		-0.16 (-0.25; -0.07)	-0.2 (-0.24; -0.17)
VLDL-TG		-0.43 (-0.52; -0.35)	-0.32 (-0.36; -0.28)
VLDL-c		-0.35 (-0.44; -0.26)	-0.2 (-0.24; -0.17)
VLDLd		-0.44 (-0.52; -0.35)	-0.32 (-0.36; -0.28)
Valine		-0.28 (-0.36; -0.19)	-0.34 (-0.37; -0.3)
Viscosity		-0.15 (-0.24; -0.05)	-0.13 (-0.17; -0.09)
XL-HDL		0.44 (0.35; 0.52)	0.36 (0.32; 0.39)
XL-VLDL		-0.41 (-0.5; -0.33)	-0.28 (-0.31; -0.24)
XS-VLDL		-0.18 (-0.28; -0.09)	-0.13 (-0.17; -0.09)
XXL-VLDL		-0.36 (-0.45; -0.27)	-0.24 (-0.27; -0.2)

Supplementary table 4B – Mendelian randomization estimates for each contributing study

	PEL82	BWHHS	WHII	CaPS	UKCTOCS	ALSPAC-M	Metabolomics consortium
Metabolite	Mean SD units (95% CI)	Mean SD units (95% CI)	Mean SD units (95% CI)	Mean SD units (95% CI)	Mean SD units (95% CI)	Mean SD units (95% CI)	Mean SD units (95% CI)
3-OH-butyrate		0.07 (-0.19; 0.33)	-0.02 (-0.22; 0.18)	0.13 (-0.18; 0.44)	-0.01 (-0.29; 0.27)	-0.04 (-0.22; 0.15)	0.02 (-0.05; 0.1)
Acetate		0.24 (-0.01; 0.5)	0.12 (-0.08; 0.32)	-0.14 (-0.45; 0.17)	0.29 (0.01; 0.56)	0.01 (-0.17; 0.2)	0.03 (-0.04; 0.1)
Acetoacetate		0.11 (-0.15; 0.37)	-0.13 (-0.33; 0.07)		-0.22 (-0.51; 0.07)	0 (-0.18; 0.19)	-0.02 (-0.1; 0.06)
Alanine		-0.03 (-0.29; 0.23)	0.06 (-0.14; 0.26)	0.08 (-0.24; 0.39)	-0.04 (-0.33; 0.25)	0.07 (-0.11; 0.26)	0.02 (-0.06; 0.09)
Albumin		-0.02 (-0.27; 0.24)	-0.02 (-0.22; 0.18)	-0.2 (-0.51; 0.11)	0.23 (-0.06; 0.52)	0.12 (-0.06; 0.3)	0 (-0.09; 0.08)
ApoA-I		0.27 (0.01; 0.53)	0.17 (-0.03; 0.37)	-0.31 (-0.62; 0)	0.16 (-0.13; 0.45)	0.06 (-0.12; 0.24)	0 (-0.09; 0.08)
ApoB		0.02 (-0.24; 0.28)	0.15 (-0.05; 0.35)	0.08 (-0.23; 0.4)	0.29 (-0.01; 0.59)	-0.07 (-0.26; 0.11)	-0.01 (-0.09; 0.07)
CLA			0.34 (0.14; 0.54)	1.22 (0.19; 2.25)		-0.27 (-0.46; -0.08)	
CRP	0.03 (-0.16; 0.21)	0.26 (-0.01; 0.53)	-0.12 (-0.32; 0.07)	0.08 (-0.3; 0.46)		-0.04 (-0.23; 0.14)	
Citrate		-0.1 (-0.37; 0.17)	-0.07 (-0.27; 0.14)	-0.25 (-0.56; 0.06)	-0.19 (-0.48; 0.1)	0.02 (-0.17; 0.21)	-0.05 (-0.12; 0.03)
Creatinine		-0.25 (-0.54; 0.04)	0.21 (0.01; 0.42)	-0.3 (-0.61; 0.01)	0.17 (-0.14; 0.47)	-0.11 (-0.29; 0.08)	-0.03 (-0.1; 0.05)
DAG			0.16 (-0.04; 0.37)	-0.28 (-0.6; 0.04)		-0.12 (-0.32; 0.07)	
DBP	0.06 (-0.12; 0.24)	-0.18 (-0.43; 0.08)	0.02 (-0.18; 0.22)	0.12 (-0.19; 0.44)		0 (-0.18; 0.18)	
DHA		0.33 (0.07; 0.59)	0.32 (0.12; 0.52)	-0.19 (-0.5; 0.13)	0.37 (0.09; 0.65)	-0.04 (-0.23; 0.15)	-0.01 (-0.11; 0.09)
Esterified cholesterol		0.16 (-0.1; 0.42)	0.16 (-0.05; 0.36)	-0.06 (-0.38; 0.25)	0.44 (0.14; 0.74)	-0.11 (-0.3; 0.07)	0.04 (-0.05; 0.14)
FAw3		0.36 (0.1; 0.62)	0.26 (0.06; 0.46)	-0.14 (-0.45; 0.18)	0.47 (0.18; 0.75)	-0.14 (-0.32; 0.05)	0.01 (-0.09; 0.11)
FAw6		0.19 (-0.07; 0.46)	0.17 (-0.03; 0.37)	-0.13 (-0.45; 0.18)	0.36 (0.06; 0.65)	-0.04 (-0.23; 0.15)	0.04 (-0.06; 0.14)
Fibrinogen		-0.14 (-0.4; 0.12)	-0.05 (-0.25; 0.15)	-0.3 (-0.67; 0.08)			
Free cholesterol		0.13 (-0.14; 0.39)	0.15 (-0.06; 0.35)	-0.13 (-0.44; 0.18)	0.29 (-0.01; 0.59)	-0.15 (-0.34; 0.04)	0.01 (-0.09; 0.11)
Glucose	0.15 (-0.04; 0.33)	0.04 (-0.22; 0.3)	0.03 (-0.16; 0.22)	-0.21 (-0.52; 0.1)	-0.09 (-0.41; 0.23)	0.23 (0.05; 0.41)	-0.06 (-0.13; 0.01)
Glutamine		0.05 (-0.21; 0.31)	-0.28 (-0.48; -0.08)	0.11 (-0.21; 0.43)	-0.01 (-0.3; 0.29)	0.07 (-0.12; 0.25)	0.03 (-0.05; 0.1)
GlycA		0 (-0.26; 0.26)	0.18 (-0.02; 0.38)	0.04 (-0.28; 0.35)	0.22 (-0.06; 0.5)	0.05 (-0.13; 0.23)	-0.07 (-0.15; 0.01)
Glycerol		-0.07 (-0.34; 0.19)	-0.01 (-0.21; 0.2)		-0.02 (-0.32; 0.28)		0 (-0.08; 0.08)
Glycine		-0.17 (-0.43; 0.08)	-0.11 (-0.31; 0.1)		-0.13 (-0.42; 0.16)		-0.03 (-0.11; 0.06)
HDL-TG		-0.2 (-0.46; 0.06)	0.2 (0; 0.4)	-0.02 (-0.33; 0.29)	0.14 (-0.15; 0.44)	-0.09 (-0.28; 0.1)	
HDL-c	0 (-0.18; 0.19)	0.23 (-0.03; 0.49)	0.1 (-0.1; 0.31)	-0.35 (-0.66; -0.04)	0.03 (-0.26; 0.32)	0.05 (-0.13; 0.23)	0.07 (-0.01; 0.15)
HDL2-c		0.26 (0; 0.52)	0.1 (-0.1; 0.31)	-0.35 (-0.67; -0.04)	0 (-0.29; 0.29)	0.06 (-0.12; 0.25)	
HDL3-c		-0.01 (-0.28; 0.25)	0.06 (-0.14; 0.26)	-0.21 (-0.52; 0.11)	0.31 (0.02; 0.6)	-0.08 (-0.27; 0.1)	
HDLd		0.22 (-0.05; 0.48)	0.1 (-0.1; 0.31)	-0.2 (-0.51; 0.11)	-0.06 (-0.34; 0.22)	0.06 (-0.13; 0.24)	-0.03 (-0.11; 0.05)
HbA1c	-0.1 (-0.29; 0.08)	-0.22 (-0.49; 0.04)					
Histidine		0.19 (-0.06; 0.45)	-0.04 (-0.24; 0.17)	0.33 (0.02; 0.64)	0.12 (-0.16; 0.4)	0.06 (-0.13; 0.25)	0.03 (-0.06; 0.11)
IDL		0.05 (-0.21; 0.31)	0.1 (-0.1; 0.3)	0.05 (-0.26; 0.36)	0.35 (0.04; 0.65)	-0.11 (-0.3; 0.07)	-0.02 (-0.1; 0.06)
IL-6		-0.18 (-0.44; 0.09)	-0.06 (-0.26; 0.14)	-0.38 (-0.8; 0.03)			
Insulin		-0.21 (-0.47; 0.05)	-0.01 (-0.2; 0.18)	0.34 (-0.1; 0.78)		-0.08 (-0.27; 0.11)	
Isoleucine		-0.03 (-0.29; 0.23)	0.14 (-0.05; 0.34)	0.11 (-0.2; 0.43)	-0.07 (-0.36; 0.22)	0.04 (-0.15; 0.22)	0.01 (-0.07; 0.08)
L-HDL		0.17 (-0.09; 0.43)	0.07 (-0.13; 0.28)	-0.31 (-0.62; 0)	-0.01 (-0.29; 0.27)	0.05 (-0.13; 0.24)	-0.01 (-0.09; 0.07)
L-LDL		0.02 (-0.24; 0.29)	0.1 (-0.1; 0.31)	-0.03 (-0.34; 0.29)	0.38 (0.08; 0.68)	-0.12 (-0.31; 0.06)	-0.01 (-0.09; 0.07)
L-VLDL		-0.02 (-0.28; 0.24)	0.12 (-0.08; 0.32)	0.03 (-0.29; 0.34)	0.07 (-0.22; 0.35)	0.03 (-0.16; 0.21)	-0.04 (-0.12; 0.04)
LA		0.14 (-0.12; 0.41)	0.15 (-0.05; 0.35)	-0.1 (-0.42; 0.21)	0.26 (-0.04; 0.56)	-0.01 (-0.2; 0.18)	0.04 (-0.06; 0.14)
LDL-TG		-0.06 (-0.33; 0.21)	0.15 (-0.05; 0.35)	-0.06 (-0.38; 0.25)	0.29 (-0.01; 0.59)	-0.12 (-0.31; 0.06)	
LDL-c	-0.1 (-0.29; 0.08)	0.07 (-0.2; 0.33)	0.09 (-0.11; 0.29)	0 (-0.32; 0.31)	0.41 (0.1; 0.71)	-0.12 (-0.31; 0.06)	0 (-0.08; 0.08)
LDLd		-0.19 (-0.45; 0.07)	-0.06 (-0.26; 0.15)	0.27 (-0.04; 0.59)	-0.22 (-0.51; 0.07)	-0.08 (-0.26; 0.1)	-0.04 (-0.13; 0.04)
Lactate		-0.03 (-0.29; 0.23)	0.02 (-0.19; 0.22)	0.14 (-0.17; 0.45)	-0.02 (-0.31; 0.27)	-0.07 (-0.26; 0.11)	0.01 (-0.07; 0.08)
Leucine		-0.05 (-0.31; 0.21)	0.21 (0.01; 0.41)	0.28 (-0.03; 0.59)	-0.1 (-0.39; 0.18)	-0.01 (-0.19; 0.18)	0 (-0.07; 0.07)
M-HDL		0.19 (-0.07; 0.44)	0.09 (-0.11; 0.29)	-0.46 (-0.77; -0.15)	-0.08 (-0.38; 0.23)	0.03 (-0.16; 0.21)	0.04 (-0.04; 0.12)
M-LDL		0.02 (-0.24; 0.29)	0.1 (-0.1; 0.3)	-0.05 (-0.36; 0.27)	0.43 (0.12; 0.73)	-0.12 (-0.3; 0.07)	0 (-0.08; 0.08)
M-VLDL		-0.07 (-0.33; 0.19)	0.16 (-0.05; 0.36)	0.02 (-0.29; 0.33)	0.13 (-0.17; 0.42)	0.04 (-0.15; 0.22)	-0.03 (-0.12; 0.05)
MUFA		-0.11 (-0.37; 0.15)	0.2 (0; 0.41)	-0.04 (-0.35; 0.27)	0.28 (-0.01; 0.58)	-0.1 (-0.29; 0.09)	0.06 (-0.04; 0.16)
PUFA		0.24 (-0.02; 0.51)	0.22 (0.02; 0.42)	-0.15 (-0.46; 0.16)	0.39 (0.09; 0.69)	-0.05 (-0.24; 0.13)	
Phenilalanine		0.08 (-0.19; 0.34)	0.07 (-0.13; 0.27)	0.7 (0.39; 1.01)	0 (-0.28; 0.28)	-0.03 (-0.22; 0.16)	0.03 (-0.05; 0.1)
Phosphatidylcholines		0.2 (-0.06; 0.47)	0.2 (0; 0.41)	-0.29 (-0.61; 0.02)	0.3 (0; 0.6)	-0.06 (-0.25; 0.13)	0.08 (-0.02; 0.18)
Phosphoglycerides		0.15 (-0.11; 0.42)	0.18 (-0.03; 0.38)	-0.31 (-0.62; 0)	0.19 (-0.11; 0.48)	-0.06 (-0.25; 0.14)	0.1 (0; 0.2)
Pyruvate		-0.11 (-0.37; 0.16)	0.09 (-0.11; 0.29)		0.1 (-0.2; 0.41)	-0.07 (-0.25; 0.11)	-0.02 (-0.1; 0.06)
Remnant-c		0.02 (-0.24; 0.28)	0.15 (-0.05; 0.35)	0.14 (-0.17; 0.46)	0.28 (-0.03; 0.58)	-0.08 (-0.26; 0.11)	
S-HDL		-0.01 (-0.27; 0.25)	0.02 (-0.18; 0.22)	-0.41 (-0.72; -0.1)	0.1 (-0.2; 0.4)	-0.04 (-0.23; 0.14)	0.03 (-0.05; 0.11)
S-LDL		0.06 (-0.21; 0.32)	0.12 (-0.08; 0.32)	-0.07 (-0.38; 0.24)	0.43 (0.13; 0.73)	-0.11 (-0.29; 0.08)	-0.01 (-0.09; 0.07)
S-VLDL		-0.11 (-0.37; 0.15)	0.18 (-0.03; 0.38)	0.04 (-0.27; 0.35)	0.2 (-0.1; 0.49)	0.01 (-0.17; 0.2)	0.01 (-0.07; 0.09)
SBP	0.09 (-0.09; 0.28)	0.06 (-0.2; 0.32)	0 (-0.2; 0.19)	-0.22 (-0.53; 0.1)		0.03 (-0.15; 0.21)	
SFA		0.1 (-0.16; 0.37)	0.19 (-0.01; 0.39)	-0.04 (-0.35; 0.27)	0.29 (-0.01; 0.59)	-0.11 (-0.3; 0.08)	
Sphingomyelins		0.22 (-0.04; 0.49)	0.09 (-0.12; 0.29)	0.15 (-0.16; 0.46)	0.32 (0.02; 0.62)		-0.02 (-0.11; 0.08)
TotFA		0.07 (-0.19; 0.33)	0.22 (0.02; 0.43)	-0.09 (-0.4; 0.22)	0.34 (0.04; 0.64)	-0.11 (-0.3; 0.08)	0.06 (-0.04; 0.16)
Total cholesterol	-0.04 (-0.23; 0.14)	0.16 (-0.1; 0.42)	0.16 (-0.04; 0.36)	-0.03 (-0.34; 0.29)	0.4 (0.1; 0.7)	-0.1 (-0.28; 0.09)	0.03 (-0.05; 0.12)
Total cholines		0.22 (-0.04; 0.48)	0.16 (-0.05; 0.36)	-0.27 (-0.59; 0.04)	0.34 (0.05; 0.63)	-0.08 (-0.27; 0.11)	
Triglycerides	0.04 (-0.14; 0.23)	-0.07 (-0.33; 0.19)	0.15 (-0.05; 0.36)	0.01 (-0.3; 0.32)	0.15 (-0.14; 0.44)	-0.01 (-0.19; 0.17)	-0.01 (-0.09; 0.07)
Tyrosine		-0.2 (-0.47; 0.07)	0.06 (-0.14; 0.26)	0.38 (0.07; 0.69)	-0.12 (-0.41; 0.17)	-0.02 (-0.21; 0.17)	0.07 (-0.01; 0.14)
VLDL-TG		-0.04 (-0.3; 0.22)	0.14 (-0.07; 0.34)	0.03 (-0.28; 0.34)	0.1 (-0.18; 0.39)	0.04 (-0.14; 0.22)	
VLDL-c		-0.03 (-0.29; 0.23)	0.19 (-0.02; 0.39)	0.18 (-0.14; 0.49)	0.21 (-0.09; 0.5)	-0.03 (-0.22; 0.15)	
VLDLd		-0.08 (-0.33; 0.18)	0.04 (-0.16; 0.24)	-0.02 (-0.34; 0.29)	-0.04 (-0.32; 0.24)	0.09 (-0.1; 0.27)	-0.01 (-0.09; 0.07)
Valine		-0.15 (-0.41; 0.11)	0.11 (-0.08; 0.31)	0.14 (-0.18; 0.45)	-0.16 (-0.45; 0.14)	0.03 (-0.15; 0.22)	-0.02 (-0.09; 0.06)
Viscosity		-0.23 (-0.5; 0.05)	0.03 (-0.16; 0.23)				
XL-HDL		0.19 (-0.08; 0.45)	0.17 (-0.03; 0.37)	-0.17 (-0.48; 0.14)	0.04 (-0.25; 0.33)	0.06 (-0.12; 0.24)	-0.03 (-0.12; 0.05)
XL-VLDL		-0.05 (-0.31; 0.22)	0.12 (-0.08; 0.32)	0.1 (-0.22; 0.41)	0.01 (-0.27; 0.28)	0.03 (-0.15; 0.21)	0.01 (-0.07; 0.09)
XS-VLDL		-0.03 (-0.29; 0.23)	0.13 (-0.07; 0.33)	0.08 (-0.23; 0.39)	0.31 (0.01; 0.62)	-0.07 (-0.25; 0.11)	-0.01 (-0.09; 0.07)
XXL-VLDL		-0.07 (-0.33; 0.2)	0.14 (-0.06; 0.34)	0.13 (-0.18; 0.44)	-0.07 (-0.35; 0.22)	0 (-0.18; 0.19)	0.01 (-0.07; 0.09)

Supplementary table 5 – Heterogeneity estimates (I^2) for meta-analysis of study-specific multivariable (MV) and Mendelian randomization (MR) estimates

Metabolite	Overall		Females		Males		European studies only		Low risk individuals only	
	MV	MR	MV	MR	MV	MR	MV	MR	MV	MR
XXL-VLDL	85	0	82	7	NA	0	85	0	78	34
XL-VLDL	88	0	91	0	NA	0	88	0	79	0
L-VLDL	86	0	84	0	NA	11	86	0	82	0
M-VLDL	88	0	68	35	NA	52	88	0	83	0
S-VLDL	81	0	0	52	NA	63	81	0	79	19
XS-VLDL	0	23	0	73	NA	49	0	23	23	48
IDL	0	36	0	78	NA	44	0	36	23	57
L-LDL	0	43	0	78	NA	64	0	43	20	65
M-LDL	0	51	0	79	NA	63	0	51	32	71
S-LDL	0	53	0	78	NA	71	0	53	40	73
XL-HDL	66	26	88	0	NA	84	66	26	19	0
L-HDL	72	22	83	0	NA	81	72	22	59	28
M-HDL	0	57	0	0	NA	86	0	57	62	58
S-HDL	0	36	0	8	NA	63	0	36	4	50
VLDLd	83	0	26	0	NA	0	83	0	73	0
LDLd	0	25	0	0	NA	50	0	25	0	66
HDLd	75	18	64	32	NA	80	75	18	0	0
Total cholesterol	94	44	71	73	96	73	0	49	92	63
Remnant-c	87	23	0	73	NA	36	87	23	76	52
VLDL-c	89	4	0	61	NA	26	89	4	81	38
LDL-c	91	45	61	71	94	73	0	47	89	66
HDL-c	75	33	90	0	0	68	76	42	73	16
HDL2-c	74	57	70	0	NA	83	74	57	72	34
HDL3-c	0	45	93	43	NA	60	0	45	0	52
Esterified cholesterol	0	56	0	80	NA	77	0	56	0	68
Free cholesterol	43	46	31	77	NA	85	43	46	27	73
ApoA-I	0	55	79	0	NA	88	0	55	0	46
ApoB	86	20	0	73	NA	56	86	20	81	56
Triglycerides	73	0	91	39	48	50	80	0	70	0
Phosphoglycerides	0	48	58	48	NA	91	0	48	0	62
Phosphatidylcholines	0	58	41	62	NA	92	0	58	0	75
Total cholines	0	67	29	73	NA	90	0	67	0	74
Sphingomyelins	30	43	0	85	NA	0	30	43	0	45
DAG	NA	70	NA	0	NA	86	NA	70	NA	84
HDL-TG	0	47	75	44	NA	76	82	0	79	0
VLDL-TG	84	49	60	22	NA	36	84	49	68	40
LDL-TG	82	0	19	68	NA	78	0	47	42	56
TotFA	0	48	5	72	NA	86	0	48	42	68
SFA	0	45	0	65	NA	76	0	45	0	61
MUFA	91	43	91	61	NA	77	91	43	77	55
PUFA	32	64	45	79	NA	89	32	64	0	66
FAw6	10	42	35	77	NA	86	10	42	22	54
LA	0	0	16	70	NA	83	0	0	53	3
FAw3	47	79	13	82	NA	84	47	79	60	80
DHA	0	76	0	64	NA	89	0	76	16	80
CLA	NA	92	NA	0	NA	46	NA	92	NA	93
Glucose	0	54	46	0	57	49	21	51	34	39
HbA1c	62	0	11	0	NA	NA	NA	NA	21	38
Insulin	0	38	0	0	NA	53	0	38	37	0
Lactate	0	0	0	0	NA	0	0	0	0	0
Pyruvate	0	0	0	24	NA	NA	0	0	0	27
Citrate	69	0	0	29	NA	0	69	0	0	0
Glycerol	74	0	83	0	NA	NA	74	0	0	0
Alanine	65	0	62	0	NA	0	65	0	58	0
Glutamine	0	43	0	0	NA	75	0	43	0	58
Glycine	87	0	71	0	NA	NA	87	0	69	55
Isoleucine	0	0	0	0	NA	0	0	0	0	9
Leucine	0	34	0	0	NA	0	0	34	0	52
Valine	45	0	87	0	NA	0	45	0	0	0
Phenilalanine	32	72	30	0	NA	91	32	72	0	79
Tyrosine	0	48	40	0	NA	54	0	48	0	42
Histidine	0	10	0	0	NA	84	0	10	0	0
Acetoacetate	0	0	0	14	NA	NA	0	0	0	0
Acetate	0	31	0	35	NA	64	0	31	74	0
3-OH-butyrate	54	0	0	0	NA	0	54	0	0	0
Albumin	0	11	0	0	NA	0	0	11	0	26
Creatinine	0	61	0	49	NA	85	0	61	22	71
CRP	0	28	81	13	41	0	0	45	0	0
Fibrinogen	16	0	87	0	NA	60	16	0	0	17
IL-6	0	3	69	0	NA	57	0	3	0	0
GlycA	0	43	0	0	NA	39	0	43	58	0
Viscosity	0	56	0	0	NA	NA	0	56	0	0
SBP	0	0	28	11	44	0	0	0	0	0
DBP	68	0	72	36	0	0	83	0	0	0

NA: not applicable (estimates from only one study available). XXL: extremely large, XL: very large, L: large, M: medium, S: small, XS: very small, VLDL: very low-density lipoprotein, LDL: low-density lipoprotein, IDL: intermediate-density lipoprotein, HDL: high-density lipoprotein, c: cholesterol, DAG: diglycerides, TG: triglycerides, TotFA: total fatty acids, SFA: saturated fatty acid, MUFA: monounsaturated fatty acid, PUFA: polyunsaturated fatty acids, FAw6: omega-6 fatty acid, LA: linoleic acid, FAw3: omega-3 fatty acid, DHA: docosaexaenoic acid, CLA: conjugated linoleic acids, HbA1c: glycated haemoglobin, CRP: c-reactive protein, IL-6: interleukin-6, GlycA: glycoprotein acetyls, SBP: systolic blood pressure, DBP: diastolic blood pressure.

Supplementary table 6. P-values for the association of demographic and lifestyle variables with SNPs selected for Mendelian randomization analysis for each participating study

	PEL82	BWHHS	WHII	CaPS	UKCTOCS case-control*	ALSPAC-M
	P-value					
Sex (male vs female)						
rs6810075	0.15	—	0.67	—	—	—
rs16861209	0.12	—	0.45	—	—	—
rs17366568	0.36	—	0.84	—	—	—
rs3774261	0.35	—	0.63	—	—	—
Age (years)						
rs6810075	0.78	0.75	0.28	0.36	0.59	0.001
rs16861209	0.56	0.58	0.27	0.01	0.57	0.83
rs17366568	0.22	0.83	0.47	0.56	0.58	0.93
rs3774261	0.68	0.03	0.96	0.87	0.43	0.15
European (yes vs no)						
rs6810075	0.06	0.50	0.48	—	0.70	0.41
rs16861209	0.75	—	0.12	—	0.16	0.35
rs17366568	0.45	0.61	—	—	0.62	0.19
rs3774261	0.44	0.95	—	—	0.85	—
Smoking (yes vs no)						
rs6810075	0.64	0.22	0.77	0.48	—	0.11
rs16861209	0.57	0.37	0.87	0.48	—	0.24
rs17366568	0.45	0.62	0.44	0.77	—	0.90
rs3774261	0.52	0.08	0.90	0.37	—	0.92
Body mass index (kg/m^2)						
rs6810075	0.63	0.49	0.49	0.21	0.05	0.45
rs16861209	0.39	0.41	0.59	0.65	0.20	0.72
rs17366568	0.47	0.73	0.87	0.11	0.66	0.32
rs3774261	0.48	0.65	0.41	0.44	0.78	0.04

ALSPAC-M: The Avon Longitudinal Study of Children and Parents – mothers' cohort; BWHHS: British Women's Heart and Health Study; CaPS: The Caerphilly Prospective Study; PEL82: 1982 Pelotas Birth Cohort; UKCTOCS: case-control study nested in The United Kingdom Collaborative Trial of Ovarian Cancer Screening; WHII: Whitehall-II Study.
